# A Tympanic Piezo‐Bioreactor Modulates Ion Channel‐Associated Mechanosignaling to Stabilize Phenotype and Promote Tenogenesis in Human Tendon‐Derived Cells.

**DOI:** 10.1002/advs.202405711

**Published:** 2024-10-22

**Authors:** Marc A. Fernandez‐Yague, Matteo Palma, Syed A. M. Tofail, Maeve Duffy, Leo R. Quinlan, Mathew J. Dalby, Abhay Pandit, Manus J. Biggs

**Affiliations:** ^1^ CÚRAM SFI Research Centre for Medical Devices University of Galway Galway H91W2TY Ireland; ^2^ Department of Chemistry Queen Mary University of London Mile End Road London E1 4NS United Kingdom; ^3^ Department of Physics and Bernal Institute University of Limerick Limerick V94 T9PX Ireland; ^4^ Centre for the Cellular Microenvironment, School of Molecular Biosciences, The Advanced Research Centre University of Glasgow 11 Chapel Lane Glasgow G11 6EW United Kingdom

**Keywords:** BNNT, electromechanical, FAK, focal adhesions, mechanotransduction, piezoelectricity, PVDF‐TrFE, tendon

## Abstract

Preserving the function of human tendon‐derived cells (hTDCs) during cell expansion is a significant challenge in regenerative medicine. In this study, a non‐genetic approach is introduced to control the differentiation of hTDCs using a newly developed tympanic bioreactor. The system mimics the functionality of the human tympanic membrane, employing a piezoelectrically tuned acoustic diaphragm made of polyvinylidene fluoride‐co‐trifluoroethylene and boron nitride nanotubes. The diaphragm is vibrationally actuated to deliver targeted electromechanical stimulation to hTDCs. The results demonstrate that the system effectively maintains the tendon‐specific phenotype of hTDCs, even under conditions that typically induce nonspecific differentiation, such as osteogenesis. This stabilization is achieved by modulating integrin‐mediated mechanosignaling via ion channel‐regulated calcium activity, potentially by TREK‐1 and PIEZO1, yet targeted studies are required for confirmation. Finally, the system sustains the activation of key differentiation pathways (bone morphogenetic protein, BMP) while downregulating osteogenesis‐associated (mitogen‐ctivated protein kinase, MAPK and wingless integrated, WNT) pathways and upregulating Focal Adhesion Kinase (FAK) signaling. This approach offers a finely tunable, dose‐dependent control over hTDC differentiation, presenting significant potential for non‐genetic approaches in cell therapy, tendon tissue engineering, and the regeneration of other mechanosensitive tissues.

## Graphical abstract

Membrane receptors such as the ion channels PIEZO1 or TREK‐1 influence transcriptional activity. Under normal physiological “electro‐mechanical” conditions, ion channel activity is regulated, limiting calcium influx and mantaining membrane potential. However, in response to injury or excesive mechanical forces, calcium activity increases byunregulated ion channel activitydeactivating tendon‐specific transcriptional activity.

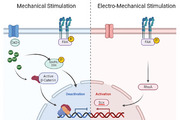

## 1 Introduction

In clinical applications, the use of tendon‐derived cells as therapeutic agents is hampered by the loss of tendon‐specific characteristics during in vitro cell expansion.^[^ ^1^, ^2^, ^3^ ^]^ Although genetic approaches, such as CRISPR/Cas9 gene editing and viral vectors for specific gene overexpression, have been explored to enhance cell therapies, these methods carry inherent risks. Off‐target effects, where unintended genomic regions are altered, can lead to unpredictable and potentially harmful consequences. Additionally, genetic approaches can trigger excessive immune responses, as the introduction of foreign genetic material can provoke the body's immune system, leading to complications that limit their clinical utility.^[^ ^4^ ^]^ Alternatively, attempts to drive cell function through in vitro manipulation of the cell physicochemical microenvironment via controlled biochemical stimulation (i.e., soluble factors, adhesive proteins) or the application of mechanical conditioning have achieved only limited success, often yielding transient effects on cell differentiation processes^[^ ^5^, ^6^ ^]^ Central to this issue is our lack of understanding of how cells sense and integrate physical and biochemicals cues into biological signals (mechanotransduction) that guide cell fate decisions^[^ ^7^, ^8^, ^9^ ^]^.

Given their multipotency and responsiveness to physical cues, mesenchymal stem cells (MSCs) are extensively studied to understand the underlying mechanisms of mechanotransduction.^[^ ^7^, ^10^ ^]^ Focal Adhesions (FAs) function as principal sites of mechanotransduction by connecting the extracellular matrix (ECM) to the actin cytoskeleton (CSK) through integrin binding.^[^ ^11^, ^12^ ^]^ In response to mechanical stress, FAs regulate their size, composition, and structure affecting signaling pathways. These changes are often accompanied by alterations in ion transport and ion channel activity, which are critical for cell adhesion, migration, differentiation, and growth.^[^ ^13^, ^14^, ^15^, ^16^, ^17^, ^18^, ^19^, ^20^, ^21^, ^22^ ^]^ Recent studies have shown that mechanosensitive ion channels (MSICs) such as PIEZO1 regulate focal adhesion dynamics and calcium entry in response to mechanical stress, directly impacting tendon cell behavior.^[^ ^10^, ^14^, ^21^ ^]^ Passini et al. have shown that PIEZO1‐mediated mechanosensitivity directly influences tendon stiffness and physical performance.^[^ ^23^ ^]^ Nakamichi et al.^[^ ^24^ ^]^ extended this knowledge by looking at functional outcomes of PIEZO1 gain‐of‐function mutations in vivo, showing superior physical performance and demonstrating the critical role of PIEZO1 in tissue mechano‐adaptation. Recent studies have identified voltage‐gated ion channels (VGICs) such as TREK‐1 are crucial mediators in the regulation of intracellular calcium dynamics, which are essential for tendon cell and MSC differentiation. Studies show that VGCCs regulate osteogenic, myogenic, and neural MSC differentiation.^[^ ^25^, ^26^, ^27^ ^]^ Ion channels contribute to the maintenance of cell functions by modulating calcium‐dependent signaling pathways critical for cellular response to mechanical stimuli.^[^ ^28^ ^]^ It is well established that low‐intensity mechanical conditioning is essential for maintaining tendon cell health, whereas excessive loading suppresses tenogenic gene expression through Wnt/β‐catenin pathway.^[^ ^29^ ^]^ Understanding how mechanotransduction influences tendon cell signaling is critical for identifying novel therapeutic targets and strategies.

Emerging mounting evidence suggests that piezoelectricity—the conversion of mechanical stress into bioelectrical signals—may act as a feedback mechanism to regulate mechanosensitivity and maintain tendon cell function. This hypothesis is supported by recent findings on the role of tendon piezoelectricity in tissue repair mechanisms.^[^ ^23^, ^24^, ^30^, ^31^, ^32^, ^33^, ^34^ ^]^ Here, we present an electromechanical stimulation (EMS) system that mimics the functionality of the human tympanic membrane (eardrum) by employing an acoustically activated piezoelectric diaphragm made of polyvinylidene fluoride‐co‐trifluoroethylene (PVDF‐TrFE), a flexible ferroelectric polymer capable of generating an electric charge in response to mechanical stress.^[^ ^35^, ^36^ ^]^ Previous studies have explored the use of PVDF‐TrFE composites with various fillers, such as BaTiO3, to enhance piezoelectric properties, which is beneficial for applications requiring high piezoelectric response.^[^ ^37^, ^38^, ^39^, ^40^, ^41^, ^42^, ^43^, ^44^ ^]^ Our study builds on this foundation by incorporating boron nitride nanotubes (BNNTs) into PVDF‐TrFE to achieve a tunable piezoelectric response suitable for biological applications.^[^ ^45^, ^46^ ^]^ Our results demonstrate oscillating EMS preserve tendon cell phenotype via ion channel modulation, potentially TREK‐1 and PIEZO1, yet further experiments are necessary to establish a direct cause‐effect relationship. Overall, our approach provides unique insights into the synergistic role of mechanical and electrical forces in hTDC culture, offering significant advancements in tendon cell expansion protocols, with broad implications for regenerative medicine. The principles underlying this bioreactor could be adapted to enhance the repair and regeneration of other mechanosensitive tissues, such as cartilage, bone, and cardiovascular tissues.

## 2 Piezo‐Bioreactor Design with Tunable Performance

**Figure 1:**
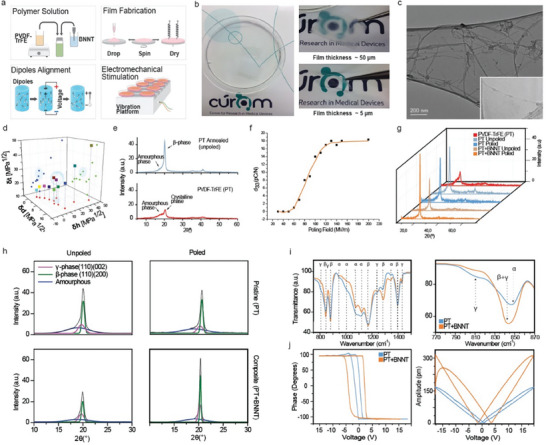
Fabrication of a Piezoelectrically Tunable PVDF‐TrFE Nanocomposite Membrane. a) Schematic illustrations of steps in fabricating PVDF‐TrFE/BNNT nanocomposite films and EMS using spin‐coating. A PVDF‐TrFE polymeric solution is applied onto a glass substrate, rapidly spun, and air‐dried to create a uniform thin film. The combination of centripetal forces and surface tension ensures a homogenous coating. b) The production of thin films with a 5–10 µm thickness after 30 s at 1000 RPMs is depicted, where the process is repeated three times to obtain films with an average thickness of 25 ± 5 µm. c) TEM image of aggregated BNNTs attributed to the polarity of the B─N bond. d) The Hansen parameters are graphically represented for different solvent and co‐solvent systems, denoted by coordinates of dispersion (δd), polar (δp), and hydrogen (δh) bonding. The sphere illustrates the Hansen parameters of BNNTs, which achieved a stable dispersion in the solution for over 24 h. e) Increased polar crystallinity, as observed in electrically poled and thermally annealed films at 132 °C for 2 h, is indicated by XRD analysis. f) Depiction of the d_33_ coefficient increases as a function of the poling field in PVDF‐TrFE/BNNT films. g) XRD analyses of pristine and nanocomposite samples before and after poling. h) XRD curves illustrate the quantification of polar and non‐polar phase fractions. i) Representative FTIR spectra showing the peaks and associated phase identification (*n* = 3, *r* = 2). j) Representative out‐of‐plane hysteresis loops demonstrating successful incorporation of BNNTs and piezoelectric behavior (*n* = 3, *r* = 6). P.T. refers to PVDF‐TrFE. Unpoled samples showed no hysteresis or piezoresponse.

Our piezobioreactor system design incorporates a multi‐functional piezoelectric acoustic membrane with adjustable mechanical and piezoelectric properties activated by a nano‐vibrational stimulation system previously described.^[^ ^10^, ^47^, ^48^, ^49^ ^]^ (**Figure** **1**a). Our prior research used a short‐distance electrospinning technique with polyvinylidene fluoride trifluoroethylene (PVDF‐TrFE) to develop a material that yielded a large transverse piezoelectric stress constant *d_31_* ^[^ ^50^ ^]^ For the present study however, we used spin coating to deposit a structurally uniform film (≈25 µm thickness, Figure 1b; Figure S1a, Supporting Information) with a high longitudinal piezoelectric stress constant *d_33,_* upon which hTDCs could be cultured following surface functionalization with fibronectin.

Previous studies have explored piezoelectric materials for tendon‐related applications, including the natural piezoelectricity of collagen fibrils,^[^ ^20^ ^]^ which plays a crucial role in tendon function, and synthetic materials like Lead Zirconate Titanate (PZT), known for its high piezoelectric response in biomedical applications.^[^ ^22^ ^]^ In our study, to tune piezoelectric performance of our system for tendon cell stimulation, a composite of PVDF‐TrFE with Boron Nitride Nanotubes (BNNTs) is used (Figure 1c). BNNTs are known for their distinct mechanical, thermal, and piezoelectric properties, with the latter arising from polarization due to the varying electronegativities of boron and nitrogen atoms.^[^ ^51^, ^52^ ^]^ We anticipated that this inherent polarization might influence the formation of electroactive crystals within the PVDF‐TrFE matrix, thus potentially enhancing its crystallinity and hence, piezoelectric performance. Integration of BNNTs into PVDF‐TrFE was challenging due to the chemical incompatibility of these materials. While BNNTs^[^ ^53^ ^]^ and PVDF‐TrFE (δt = 23.17)^[^ ^54^ ^]^ share similar dispersion and hydrogen bonding Hansen solubility parameters, the differences in their polar parameters can complicate BNNT dispersion within PVDF‐TrFE.^[^ ^55^ ^]^ Moreover, conventional BNNT dispersion methods, such as sonication and chemical modifications, might impact the nanotube electroactivity, making PVDF‐TrFE/BNNT composite formulation challenging. Initially, we encountered those dispersions tended to form extensive BNNT aggregations, likely attributed to the high polarity of the B─N bond resulting in complex inter‐tube interactions (Figure 1c). By surveying a variety of polymer coatings^[^ ^56^ ^]^ and co‐solvent systems (Figure 1d), we were able to successfully disperse and stabilize the BNNT suspensions using N,N‐dimethylacetamide (DMAc) and acetone via a gentle sonication mixing procedure that facilitates the polar interactions between BNNTs and PVDF‐TrFE (Figure S1b, Supporting Information). This approach enabled us to achieve a minimum percolation threshold (1% w/v) while preserving the inherent structure of the BNNTs (Figure 1c,d; Figure S1b,c, Supporting Information).

Consequently, we evaluated the effect BNNT incorporation on the structural and piezoelectric performance of the PVDF‐TrFE films. It is generally accepted that the piezoelectric constant (*d_3j_*) depends on the macroscopic permanent remanent polarization (*P_r_*) and the stress‐induced polarization (ΔP).^[^ ^57^, ^58^ ^]^ Ferroelectric *β* crystals of PVDF are known to possess the highest spontaneous polarization compared to ferroelectric *γ* polymorphs, which exhibit up to ≈64% of the spontaneous polarization shown by β polymorphs,^[^ ^55^ ^]^ whereas the non‐polar polymorph α of PVDF is paraelectric. Methods including thermal annealing just below the Curie temperature (Tc) and high electric field poling are employed to boost the *β* phase content (Figure 1e). It is, however, challenging to obtain films with fully aligned, compact, and pure *β*‐crystals capable of surviving high electric fields at the poling temperature. After poling using a custom‐made electrical poling rig (Figure S1d, Supporting Information) to maintain constant pressure while controlling atmospheric parameters (e.g., temperature and humidity) all ferroelectric domains within the crystals align toward the normal direction of the film, obtaining a high d_33_ piezoelectric coefficient (Figure 1f). To identify the crystal structures, present in the films, we employed X‐ray diffraction (XRD), modulated differential calorimetry (MDSC) and Fourier transform infrared (FTIR) spectroscopy (Figure 1g–i; Figure S2, Supporting Information). From the MDSC and XRD analyses, we found that annealing increased the crystallinity from ≈0.57 to ≈0.63 in pristine and to ≈0.69 in composite PVDF‐TrFE formulations (Figure 1g–h). Using quantitative FTIR analysis, we determined that β crystallinity (fβ) increased from ≈0.72 to ≈0.74 and ≈0.81 in pristine and composite films, respectively (Figure 1i and **Table** **1**). While the overall crystallinity remained unchanged for both samples post‐poling, we noticed the presence of γ crystals in the pristine samples.

**Table 1:** Structural, mechanical, and piezoelectric properties of poled and unpoled (thermally annealed) films.

Sample	Annealing Temp/T_c_ [°C]	*χ*_c_ [%]	EA Fraction *β* + *γ*	*β*‐crystal size [nm]	Piezoelectric Strain Constant [*d_33_*_,_ pC N^−1^]	Elastic Modulus [*c*, MPa]	Resilience Modulus [MPa]	Toughness Modulus [MPa]	Polarisation Modulus [*P_m_*, mC m^−2^]
PT (as received)	RT	57	0.55	8.34	–	–	–	–	–
PT (unpoled)	120	64	0.72	17.29	0	–	–	–	–
PT (poled)	120	65	0.74	24.39	15.40	801	66	3307	12
PT+BNNT (unpoled)	132	69	0.79	19.13	0	–	–	–	–
PT+BNNT (poled)	132	69	0.81	33.02	19.30	1009	112	988	19

In contrast, composite films were predominantly characterized by β crystals. These findings imply that the application of a high electric field during poling has the effect of transforming all α crystals into either β or γ crystals. Intriguingly, we noticed that poling composite films led to an increase in β crystal size (from ≈17 to ≈24 and ≈33 nm for pristine and composite formulations respectively, Table 1). The higher fraction of larger β crystals in the composite PVDF‐TrFE films suggested that BNNTs interacted with the crystal formation processes (Figure S3, Supporting Information). Therefore, we performed crystal kinetic analyses (non‐isothermal and isothermal, Tables S1 and S2, Supporting Information) and we observed significant changes in the crystal kinetics and crystallization mechanisms. From this study, we inferred that 2D crystal growth occurs between the BNNTs, which act as nucleation sites and promote higher crystallization rates (Table 1). As the polymer chains start to order themselves around the BNNTs, they can continue to grow, leading to larger crystal formations.

Next, we determined how BNNT inclusion affected dipole alignment, nanoscale topography, and electrical and mechanical properties of the PVDF‐TrFE films (Figure S4, Supporting Information). Atomic force microscopy (AFM) indicated a consistent surface topography which did not significantly change with the incorporation of BNNTs into the PVDF‐TrFE polymer matrix (RMS = 12.7 ± 1.2 and 14.8 ± 2.6 nm for pristine and composite films respectively) (Figure S4d–h, Supporting Information). Importantly, the inclusion of BNNTs significantly improved the material's mechanical dissipation, as confirmed by MDSC and tensile analysis results, suggesting a strong integration and dispersion of BNNTs into the PVDF‐TrFE matrix. This is key for achieving efficient stress transfer to BNNTs, hence optimizing their piezoelectric activation.

Switching spectroscopy piezoresponse force microscopy (SS‐PFM, Figure 1j) was employed to assess changes in dipole alignment and piezoresponse in pristine and composite PVDF‐TrFE substrates. Voltage‐amplitude loops displayed diverse forms, with continuous squared loops signaling ferroelectric behavior. Broad hysteresis loops were identified in all film samples, affirming piezoelectric behavior. Notably, composite films showed elevated remnant polarization and a higher coercive field (Figure 1j), indicating a greater resistance to the dipole reversal process. While lower coercive fields are commonly linked to increased crystallinity in PVDF‐TrFE due to dipole alignment in its crystal lattice,^[^ ^59^ ^]^ our composite films exhibited a slightly elevated coercive voltage. This difference is attributed to the high‐aspect ratio of the BNNTs, which not only restricts domain wall mobility but also introduces interfacial effects, thereby bolstering the material's resistance to polarization changes.

Collectively, these findings reveal that the successful integration and dispersion of BNNTs within the PVDF‐TrFE matrix results in a piezoelectric material with enhanced multifunctional properties. Despite the lower C/N values compared to commercial bulk samples, our films offer several advantages, including better integration with biological tissues and enhanced flexibility. Consequently, these findings underscore the potential for utilizing BNNT‐enhanced PVDF‐TrFE in high‐performance applications, such as EMS.

It is important to note that unpoled PVDF‐TrFE films (non‐piezoelectric, with zero macroscopic polarization) were fabricated to isolate the effect of electric cues from mechanicals on the potential cellular response. The degree of crystallinity, crystallite size, and macroscopic properties, including piezoelectric coefficients for these samples, are shown in Table 1. The optimal annealing temperatures for pristine (120 °C) and composite (132 °C) films that significantly enhanced the formation of the beta phase were determined from our previous thermal studies analyses.^[^ ^60^ ^]^

The elastic moduli of these samples are 2–3 orders of magnitude lower than that estimated from quantum mechanical simulations^[^ ^61^ ^]^ due to the presence of different PVDF crystalline phases and to the degree of material crystallinity. The recorded values compared well with the elastic modulus of commercial PVDF (≈2200 MPa), e.g., that reported in the PVDF technical properties data sheet (theplasticshop.co.uk). The addition of BNNT, significantly stiffened these samples, as expected. Longitudinal piezoelectricity in poled PT and PT‐BNNT samples compared well with commercially available bulk samples (Piezoelectric Materials | P(VDF‐TRFE) | Arkema Piezotech). The polarisation modulus *P_m_* (C/m2) can be found using:

(1) $$P_{m}=d.c$$

where d is the piezoelectric strain constant (C N^−1^) and c is the elastic modulus (N m^−2^). From Table 1 we can derive Pm for poled PT+BNNT to be 19 mC m^−2^, almost 1.5 times higher than that measured in poled PT (≈12 mC m^−2^). Both values are somewhat lower yet consistent with the remnant polarization values reported in commercial samples (45–85 mC m^−2^).^[^ ^62^ ^]^ The higher polarization modulus of PT‐BNNT formulations indicates a relatively higher charge density over a given area indicating the potential of these materials for cell culture experiments.^[^ ^63^ ^]^ While PVDF‐TrFE/BaTiO_3_ composites have been shown to achieve higher piezoelectric strain constants, our composite films exhibit a balance between piezoelectric performance and mechanical flexibility, more suitable for vibrational stimulation.

**Figure 2:**
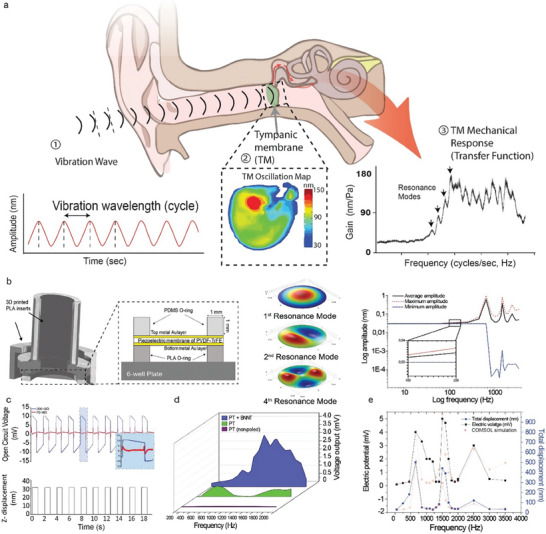
Piezobioreactor Multi‐Resonant Configuration for Tunable Piezoelectric Performance and Frequency Selectivity of Mechanically Actuated Acoustic Diaphragm. Schematic representation of tympanic membrane function a): Vibration‐wave, depicted as alternating high‐and low‐pressure waves, impact the tympanic membrane, causing it to resonate. This vibration transfers energy through the middle ear as mechanical amplifications, represented by the gain transfer function graph. The inset shows the oscillation map of the tympanic membrane under stimulation. b) Schematic representation of the piezobioreactor diaphragm configuration, with the membrane tension precisely modulated for an optimal vibrational response. COMSOL modeling output showcasing the harmonic response of the membrane within the frequency range of 0–2000 Hz at a set actuator amplitude of 30 nm. Below ≈700 Hz, the membrane response remains in phase with the input vibration, as detailed in the magnified inset (100–200 Hz). Beyond this frequency, resonant modes appear (≈700 Hz, ≈1500 Hz, and ≈2500 Hz), leading to significant amplification and deformation of the membrane, as depicted by the model's minima and maxima amplitude data. c) Sample electrical responses as captured by voltameters with two distinct inner impedances (70 MΩ and 200 GΩ) (top) juxtaposed against the corresponding input signal (bottom). d) Voltage output as a frequency sweep function for unpoled pristine, poled pristine, and poled composite PVDF‐TrFE films. e) Total displacement and electric potential as a function of frequency sweep, obtained through scanning laser interferometry‐based voltammetry; the data points represent actual measurements, while the curves represent COMSOL modeling outputs (*n* = 7 samples).

Our system, designed to resonate at kHz frequencies, mimics the natural mechanism of the tympanic membrane that converts vibrational energy into mechanical signals (tympanum, **Figure** **2**a).^[^ ^64^, ^65^ ^]^ The bio‐inspired system's core comprises a diaphragm film suspended above an aperture within a unique cell culture vessel (Figure 2b). This configuration is essential for enabling the film's free oscillation, thereby inducing a resonant effect. This design serves to amplify (gain) acoustic waves, effectively replicating the auditory transduction process.

The resonant behavior of the film markedly enhanced its vibrational response (Figure 2b), thereby optimizing the transduction of acoustic energy into electrical signals at specific resonant frequencies, closely associated with the membrane's mechanical impedance (size of the aperture and the inherent mechanical properties of the film). In our downscaled system, the relationship between the resonant frequency and the film's mechanical impedance adheres to an inverse proportionality, as expressed in:

(2) $$f_{r}\alpha \frac{h}{d^{2}}\sqrt{E}$$

where *f_r_* is the resonance frequency, *d* is the diameter of the aperture, and *h* and *E* are the thickness and specific elastic modulus of the film, respectively.^[^ ^66^ ^]^ Using Multiphysics COMSOL simulations, we calculated the film's mechanical resonance frequency and electrical output. This was then validated by measuring the open‐circuit voltage outputs of the actuated films across a range of frequencies (0.1–4 kHz, Figure 2; Figure S5, Supporting Information). It's worth noting that the first resonant eigen frequency was observed to increase correspondingly with the diaphragm tension, a relationship proportional to the square root of the tension (Figure S6, Supporting Information). However, adding a liquid phase above the vibrating film (required for cell culture) dampened both the film displacement amplitude and the resonant frequency. This effect was due to the in‐phase movement of the fluid with the vibrating film and could be represented by an added virtual mass incremental (AVMI) factor Figures S7 and S8, Tables S3–S5, Supporting Information). Importantly, interactions between the film and fluid were necessary for resonant peaks to occur. Following stabilization in our model, the vibrating films exhibited resonance peaks at ≈700 Hz and 1500 Hz (Figure 2b). We observed that the high film loss factor (loss factor ≈ 0.2) was able to cover a large frequency range (0.1–3 kHz) by broadening the resonant bandwidth of the system (Δf = 400 Hz). It can hence be understood that the thin polymer film with elastic damping and a low Q factor (≈18,) exhibited increased oscillation around the resonance frequencies (Figure S7, Supporting Information).

To accurately assess the piezoelectric performance of the films, we employed an advanced acquisition system with an input impedance exceeding that of the film's electrical impedance, which is defined by its effective capacitor impedance and loading frequency. This adjustment mitigated signal attenuation, enabling precise open‐circuit voltage measurement. We compared two different acquisition systems where the film was subjected to a 1 Hz square wave input (Figure 2c): one with an internal resistance of 70 MΩ and another with 200 GΩ. Under the 70 MΩ resistance system, we observed a noticeable drop in output voltage amplitude (sub‐5 mV) with rapid decay within 100 ms. Conversely, the 200 GΩ resistance system demonstrated a consistently square wave at a 1 Hz cycle, exhibiting a gradual slow decay. This contrast highlights the dependency of the measured output voltage on the voltmeter's internal resistance, challenging the prevalent assumption of measurement independence from instrumentation. This issue arises from mistakenly assuming the resistance of the acquisition systems is infinite, yet practically, finite resistance facilitates charge flow, which leads to directional shifts in response to strain variations, as demonstrated in.^[^ ^67^, ^68^ ^]^ Additionally, we incorporated a Fast Fourier Transform (FFT) band‐pass filter to eliminate background noise and interference from proximal equipment (Figure S8, Supporting Information), ensuring a more accurate evaluation of the electrical outputs (Figure 2d). The filtered signals at the vibration frequency closely mirrored the unfiltered signals, efficiently isolating extraneous frequency contributions. In our experiment, we probed the resonance's effect on voltage output. This was done by maintaining a fixed vibration displacement amplitude (≈30 nm) of the film actuator while varying the frequency. As shown in Figure 2d, the actuator frequency significantly influenced the diaphragm voltage output. Peak outputs were recorded at ≈700 Hz (≈20 µV nm^−1^) and ≈1500 Hz (≈180 µV nm^−1^), corresponding to the first and second resonance peaks of the composite membrane. Conversely, unpoled control membranes, demonstrated negligible electrical voltage with a constant sensitivity of ≈1.3 µV nm^−1^.

Unsurprisingly, the size of the aperture significantly influenced the output voltage, since only ≈10% of the voltage was obtained when a non‐resonant configuration was used. This relationship demonstrated a quadratic dependence on the aperture diameter (Figure S8, Supporting Information). In line with our COMSOL model projections, sensitivity escalated toward resonance when the frequency surpassed 200 Hz, marked by a substantial variance in output (Figure 2b). Maintaining a consistent film thickness of 25 µm, we identified the optimal sensitivity at an aperture diameter of 12.5 mm, yielding an output of 2.5 mV (Figure S8, Supporting Information). However, there was a marked reduction in output when the aperture diameter was reduced to less than 7 mm or when it exceeded 15 mm, a trend that closely mirrored our model's predictions and supported the occurrence of resonant peaks. This synergy of biomimetic design and material innovation underlines the potential of our approach in advancing the functionality and efficiency of piezoelectric‐based bioreactors.

## 3 High‐Frequency Mechanical Stimulation Promotes Osteogenesis While Inhibiting Tenogenesis in Mesenchymal Stem Cells

Investigating the response of undifferentiated MSCs to nanoscale mechanical vibrations provides critical insights into the mechanosignaling pathways that influence cell differentiation. Previous studies have shown that vibrations, in the kHz range, can promote osteogenesis in MSCs without requiring biochemical additives.^[^ ^10^ ^]^ To explore the influence of MS on MSC differentiation, we performed high‐frequency MS for 1 and 5 days, corresponding to key phases of cell proliferation and early differentiation to observe potential shifts in lineage commitment under mechanical cues.^[^ ^10^, ^69^, ^70^ ^]^ Importantly, we used hTDCs as a control group to benchmark tendon‐specific gene expression and experiments were conducted on non‐piezoelectric films to isolate the effects of MS alone on cellular responses. Furthermore, to ensure optimal cellular attachment and viability, we modified the surface chemistry of our PVDF‐based materials by covalently attaching fibronectin (Fn). This enhanced cell viability and controlled proliferation (compared to non‐modified surfaces) of our cultures for up to 10 days, regardless of the chemistry or static polarization of the underlying material (Figure S9, Supporting Information).^[^ ^71^ ^]^

**Figure 3:**
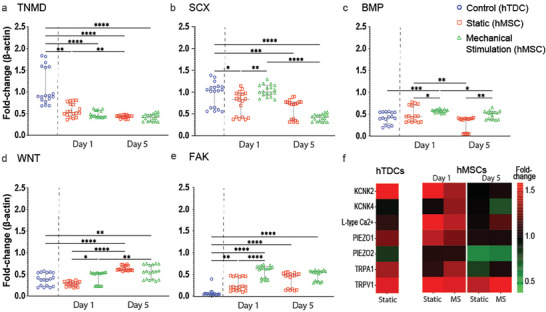
Mechanical stimulation drives osteogenic pathways while inhibiting tenogenic differentiation in MSCs. Nanoscale vibrational stimulation (MS) led to a significant downregulation in the expression of a) TNMD and, b) SCX indicating a deviation from tenogenic lineage, using tendon cells as a reference for baseline expression levels. Simultaneously, an upregulation in the expression of c) BMP and, d) WNT was observed, suggesting the onset of osteogenic differentiation. e) FAK indicates an increase in cell adhesion signaling as a response to MS. f) Ion channels expression changes response to MS at days 1 and 5. Data is represented as Median ± range (*N* = 4, *r* = 6). Statistical analysis was performed using Non‐parametric Kruskal–Wallis followed by Dunn's multiple‐comparisons test. Statistical significance is indicated as follows: ^*^ *p* < 0.05, ^**^ *p* < 0.01, ^***^ *p* < 0.005, ^****^ *p* < 0.001.

Our analysis focused on cell adhesion (e.g., FAK), tenogenesis (e.g., SCX, TNMD), and osteogenesis (e.g., BMP, WNT). We observed differential and contrasting expression patterns of markers associated with osteogenesis and tenogenesis. The results revealed a clear divergence in differentiation pathways. After 5 days of MS, osteogenic markers BMP and WNT were significantly upregulated, indicating a shift toward osteogenesis. In contrast, tenogenic markers SCX and TNMD were downregulated, suggesting that MS may inhibit tenogenesis (**Figure** **3**a–d). Further analysis revealed activation of FAK signaling, which implicates cell adhesion mechanisms are important for the observed osteogenic differentiation (Figure 3e). The osteogenic response to mechanical stimulation appears to be modulated not only by cell adhesion dynamics but also by mechanosensitive ion channels. Our data show significant increases in the expression of several key ion channels, including TRPV1, PIEZO1, KCNK2, and TRPA1, in response to vibrational stimuli (Figure 3f). The observed upregulation, in particular of PIEZO1 in response to MS could be partially attributed to the shear stress generated by the vibrating film, which was estimated to be ≈0.32 Pa. These channels are known to play crucial roles in transducing mechanical signals into biochemical responses, further supporting the hypothesis that ion channel activity, coupled with RhoA/ROCK signaling, is vital in driving the osteogenic differentiation of MSCs while inhibiting tenogenic pathways.^[^ ^48^, ^72^, ^73^, ^74^ ^]^ By comparing MSCs subjected to MS with the baseline provided by hTDC controls, our findings suggest that mechanical cues alone may favor osteogenesis over tenogenesis in undifferentiated cells. These contrasting effects observed underscore the complexity of mechanotransduction pathways for cell fate differentiation.

## 4 Mechanical Stimulation Induces Ca^2+^ Entry in Tendon Cells

Tendon tissue is subjected to cyclic mechanical loading during locomotion. This mechanical strain is bored by a highly organized matrix composed mainly of collagen type I that elicits an electrical response due to its piezoelectric properties (piezoelectric stress constant of ≈2 pC N^−1^). Therefore, the oscillating collagen fibers during locomotion create a mechanoelectrical microenvironment,^[^ ^75^, ^76^ ^]^ modulating tendon cell function. To explore how tendon cells sense mechanical forces, we designed a functional imaging system that combines fluorescence microscopy with MS. This setup enabled Ca^2+^ imaging on hTDCs labeled with Fluo‐4 AM, allowing us to observe their real‐time response to both MS and EMS.

**Figure 4:**
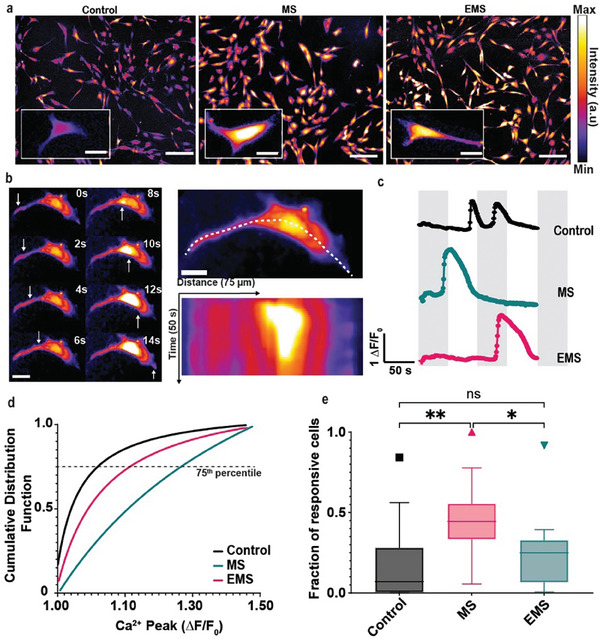
Mechanical Stimulation Modulates Ca^2+^ Events in hTDCs. a) Fluo‐4 imaging illustrates Ca^2+^ signals at baseline (Control, left), and during MS (middle) and EMS (right) both at 1500 Hz. Insets provide magnified views, emphasizing changes in Ca^2+^ intensity. Scale bars: 100 µm (inset 20 µm). b) Time‐lapse imaging (left) depicts Ca^2+^ waves originating from the cell periphery (arrows). Scale bars: 20 µm. The right panel shows a kymograph of a representative cell. c) Representative Ca^2+^ signals for Control (top), MS (middle), and EMS (bottom). d) Fraction of cells responding to stimuli (*n* = 112 cells for Control, *n* = 99 for MS, and *n* = 136 for EMS from 3 independent experiments). Tukey's multiple‐comparison test: *p* < 0.001. One‐way ANOVA test was used: ^*^ *p* < 0.05, ^**^ *p* < 0.01 and NS. Full statistics are in the Methods. g) Cumulative distribution of response amplitude (ΔF/F0) for the different stimulus. The 75th percentile Δ*F*/*F*_0_ values for each group: 1.04 (Control), 1.11 (EMS), 1.29 (MS) (*n* = 112 cells for Control, *n* = 99 for MS, and *n* = 136 for EMS from 3 independent experiments). Full statistics are in the Methods.

We observed marked differences in the calcium response of hTDCs to MS and EMS (**Figure** **4**a, calcium activity for Control, MS, and EMS). This observed dynamic calcium flux is crucial to a myriad of cellular processes, including but not limited to, mechanotransduction, resting membrane voltage regulation, and cell cycle progression.^[^ ^77^, ^78^, ^79^ ^]^ Interestingly, hTDCs under static conditions exhibited dynamic Ca^2+^ flux activity, highlighting the potential electrosensitivity of these cells (Figure 4b, time‐lapse imaging showing the progression of calcium flux across a single cell, highlighting a calcium wave that traverses the cell over a 14‐s period). To quantify this dynamic calcium activity response, we analyzed kymographs of individual cells (Figure 4b, lower panel), which provide a visual representation of the spatial and temporal characteristics of the calcium response, allowing us to measure the speed, onset time, peak intensity, and duration of the calcium waves propagating through the cell. The observed Ca^2+^ peaks persisted for an average duration of 45 ± 15 s. We noted that hTDCs typically exhibited numerous spontaneous low‐intensity Ca^2+^ peaks under static conditions (Figure 4c), whereas cells responded during both MS and EMS with single, high‐intensity, Ca^2+^ peaks.

We quantified mechanosensitivity as the cumulative distribution of Ca^2+^ peaks (ΔF/F_0_) across cells Figure 4d) and as the proportion of responsive hTDCS (Figure 4e). Significantly enhanced calcium activity was seen for MS (distribution of Ca^2+^ peaks was shifted toward larger values in Figure 4d), while electromech showed a more subdued response. The peak amplitude of transient responses was significantly higher for MS than for EMS (129% vs 111%, respectively; Figure 4d). Significantly more responsive cells were seen for MS than EMS (Figure 4e, *p* < 0.05). This indicates that MS not only amplifies the overall calcium signaling within each cell but also increases the number of responsive cells, suggesting that EMS modulates MS‐induced calcium‐activity, which may modulate the downstream signaling pathways.

Taken together, these findings suggest a mechanistic pathway where MS enhances calcium signaling, which in turn inhibits tenogenic differentiation in hTDCs, consistent with previous observations that mechanical stress can act as a barrier to tendon differentiation.^[^ ^29^ ^]^ However, EMS attenuates this calcium response and may play a crucial role in modulating the downstream signaling pathways.

## 5 Modulation of Mechanosensitive Ion Channels and Contractility Enhances Tendon Cell Differentiation

Our previous work demonstrated that high‐frequency MS (>1 kHz) efficiently promotes MSC osteogenic differentiation. In contrast, lower frequencies (e.g., 500 Hz) did not produce comparable effects.^[^ ^10^, ^47^, ^48^, ^49^ ^]^ During osteogenic differentiation at kHz frequencies, significant increases in BMP and RUNX2 expression are seen, whereas at sub‐kHz gene expression levels were not sufficiently altered to initiate differentiation. These results suggest that the frequency determined the level of mechanical stress and associated cellular response, influencing the activation of mechanotransduction pathways.^[^ ^80^ ^]^

In our study, we first examined how low frequency MS (≈700 Hz) affects hTDCs function. We chose this frequency based on the diaphragm's resonance peak spectrum, which shows a resonant peak at ≈700 Hz (Figure 2e). At this frequency, we observed a significant increase in tendon‐related markers (compared to no stimulation), suggesting that lower mechanical stress supports tenogenic differentiation (Figure S10aSupporting Information). This finding is consistent with studies showing that tenogenesis is best supported by low‐intensity stimulation, while excessive forces can have adverse effects on hTDCs.^[^ ^81^, ^82^ ^]^ Next, we explored the impact of high‐frequency MS (≈1500 Hz) on hTDCs, which resulted in a pronounced shift in tenogenic markers (D5: SCX, BGN, *p* < 0.01 Figure S10, Supporting Information) associated with significant activation of differentiation pathways (BMP, *p* < 0.05; MAPK, *p* < 0.01, Figure S11, Supporting Information), suggesting a phenotypic drift of hTDCS.

**Figure 5:**
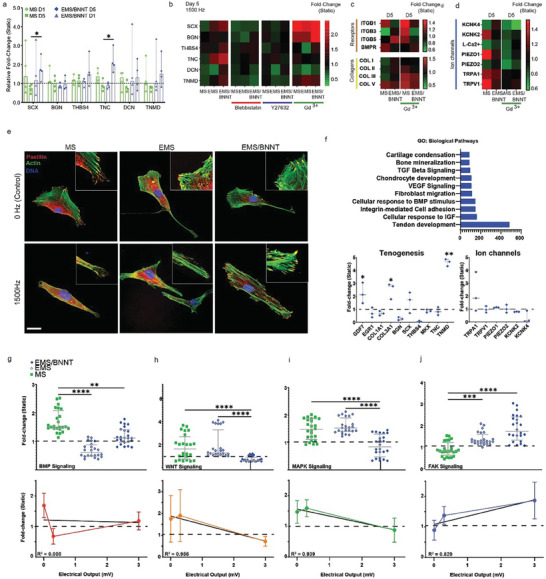
EMS Modulates hTDCs Functional Response. a) Evaluation of protein expression levels related to tendon maturation and collagen synthesis at days 1 and 5 in hTDCs cultured under MS and EMS at 1500 Hz. Data are presented as Median ± IQR (*N* = 4). b) Effect of inhibiting ROCK/RhoA (Y27623) or Myosin II activity (blebbistatin) on tendon‐specific markers expression, (*N* = 4). c) Effect of inhibiting ion channels activity (Gd3+) on collagens, integrin receptors, and d) on ion channels expression (*N* = 4). e) Paxillin expression as a marker for cell adhesion signaling (*N* = 3). f) Gene ontology pathway analysis showing the activation of specific biological pathways induced by EMS (top). Gene expression fold‐change (bottom) in cells subjected to EMS (D5, *N* = 3). g) Protein expression (top) and correlative analysis (bottom) for TGF‐β, h) WNT i) MAPK, and j) FAK activation under different EMS levels (0, 1, and 3 mV). Description of pathway activation is in the Experimental Section. Data is represented as Median ± range (*N* = 3, with the experiment replicated six times). Statistical analysis was performed using Non‐parametric Kruskal–Wallis followed by Dunn's multiple‐comparisons test. The representation of statistically significant differences is denoted as ^*^ *p* < 0.05, ^**^ *p* < 0.01, ^***^ *p* < 0.005, ^****^ *p* < 0.001.

We investigated the role of contractility (mechanical forces) using Blebbistatin (Myosin II ATPase activity) and Y‐27632 (Rho‐associated kinase) inhibitors on hTDC responses (**Figure** **5**b). Our findings demonstrated that cellular tension is required for hTDCs functional response, showing that inhibiting contractility eliminated the upregulation of tendon markers for both MS and EMS (TNC, SCX, *p *> 0.987). In stark contrast, blocking calcium influx using Gd3+ (Figure 5b) led to a marked increase in tenogenic marker expression (SCX, BGN, *p* < 0.01), collagen synthesis (Col II and III; *p* < 0.01, Figure 5c) and integrin expression (ITG1, 3, 5; *p* < 0.01, Figure 5c), with these effects being particularly pronounced in the MS group. Similarly, the application of EMS appeared to attenuate MS‐induced responses, suggesting a modulatory effect of electrical stimulation on the cellular response to mechanical cues, highlighting the potential of manipulating ion channel activity to promote tendon cell differentiation. These findings indicate that inhibiting calcium‐mediated signaling pathways can enhance tendon differentiation, likely by promoting extracellular matrix production and integrin‐mediated cell‐matrix interactions (Figure 5c). Overall, these results suggest that manipulating both cellular contractility and ion channel activity offers a promising strategy for directing hTDC differentiation. Specifically, while contractility is crucial for maintaining tenogenic differentiation under MS, inhibiting calcium influx may further enhance tendon‐specific marker expression and matrix synthesis, providing new insights into the optimization of biophysical cues for tendon cell culture.

Our results revealed a distinct and opposing effect of MS and EMS in regulating mechanotransduction pathways in hTDCs. Specifically, we detected significant differences in the expression profiles of mechanoreceptors and downstream signaling pathways.

Under MS alone, there was a marked upregulation of the mechanosensitive ion channels PIEZO1, TRPV1, and KCNK2, which corresponded with a lower expression of tendon‐related markers (Figure 5d). This suggests that MS tends to favor osteogenic differentiation, likely through the activation of these mechanosensitive pathways. This is consistent with the observed activation of the Wnt/β‐catenin and MAPK/ERK signaling pathways, both of which are known to promote osteogenesis.

In contrast, EMS elicited a different cellular response, characterized by high levels of tenogenic marker expression (SCX, THBS4, TNC, and TNMD, *p* < 0.05, Figure 5b) and reduced expression of PIEZO1, TRPV1, and TRPA1 (*p* < 0.05, Figure 5d). This indicates that EMS may promote tenogenic differentiation by modulating mechanotransduction differently than MS. The increased recruitment of paxillin to focal adhesions under EMS further supports the idea that EMS enhances tenogenic differentiation through integrin‐mediated signaling pathways (Figure 5e).

Genomic analyses corroborated these findings, showing modulation of ion channel activity (EMS, Figure 5f), which aligns with the activation of tendon‐associated pathways (Figure S12, Supporting Information; Figure 5b). The interaction between ion channel signaling and integrin‐mediated mechanotransduction appears to be a critical factor in determining cell fate under different mechanical stimuli.

Finally, we obtained that both MS and EMS activate the BMP signaling pathway at different levels, with MS showing the highest levels (1.65‐fold change with respect to static, *p* < 0.001, Figure 5g). MS promoted osteogenesis (Figure 5b) and correlated with activation of the MAPK/ERK and Wnt/β‐catenin pathways (Figure 5h,i) likely driven by the upregulation of mechanosensitive ion channels (Figure 5d,f). In contrast, EMS downregulates osteogenic pathways, promoting tenogenesis instead, likely through the ion channels modulation and enhanced integrin‐mediated FAK signaling (Figure 5j). This differential regulation highlights the potential of EMS as a tool for selectively directing tenogenic differentiation while minimizing undesired osteogenic outcomes. Overall, our study demonstrates that by fine‐tuning the mechanical environment through EMS, it is possible to optimize tendon cell culture expansion and inform future strategies for tissue tendon regeneration or prevent heterotypic ossifications.

## 6 Discussion

How tendon cells sense and integrate mechanical and electrical forces into biochemical signals is not completely understood. While previous research has primarily focused on the mechanical aspects of tendon cell differentiation and function, our findings highlight the significant role of electrical stimuli, delivered through a new piezo‐bioreactor, in modulating cellular responses.

The role of MS in tendon differentiation has been extensively studied, with findings supporting the idea that low‐magnitude, low‐frequency mechanical cues promote tenogenic differentiation. Our observations align with these studies, demonstrating that low‐frequency MS supports the maintenance of tendon‐specific markers (e.g., SCX, TNMD) and collagen synthesis. In contrast, we noted that high‐frequency MS drives hTDCs differentiation toward osteogenic lineages as seen by the upregulation of osteogenic markers (BMP, WNT pathways). Also, these results are consistent with earlier MSC research, where ion channels‐mediated mechanosensing controls cell differentiation,^[^ ^10^, ^48^ ^]^ highlighting the importance of controlled mechanical environments in regulating cell function.

Beyond mechanical stimulation, our study revealed the significant regulatory impact of electrical stimuli. Growing evidence shows that piezoelectric properties of tendons are essential for various cellular functions, including cell proliferation, differentiation, migration, and mechanosensitivity. Moreover, ion channel dysregulation and disrupted bioelectric signaling have been linked to several pathological states. However, there is still a lack of comprehensive evidence regarding how cells respond to electrical stimuli and the specific molecular mechanisms involved in this process. Herein, we developed a bioreactor using a piezoelectrically tuned diaphragm made of a piezoelectric polymer (PVDF‐TrFE) capable of delivering targeted EMS. To tune its performance, we added boron nitride nanotubes (BNNTs) to the polymeric matrix. This not only enhanced the mechanical properties (resilience), but also increased its resistance to changes in polarization (switch resistance), crucial for maintaining consistent electrical stimulation for prolonged time, as confirmed by Modulated DSC, tensile, and PFM results. Our results demonstrated that EMS effectively preserved tendon cell phenotype by modulating ion channel activity. The distinct calcium signaling patterns observed under MS and EMS in our study suggest that PIEZO1 and other ion channels like TRPV1 and KCNK2 play pivotal roles in directing cell fate.

This is further supported by the differential expression of these channels under MS and EMS, indicating that they might serve as molecular switches that modulate the downstream signaling pathways. The increase in tendon markers, collagen synthesis, and integrin expression in the MS group but not in EMS following ion channel activity inhibition by Gd^3+^ treatment, indicates that EMS might counteract or modulate the osteogenic effects induced by MS. This could be due to the distinct signaling pathways activated by electrical cues, which may interfere with or override the mechanosensitive MAPK and Wnt pathways that we observed are upregulated during MS. Future studies are needed to dissect these mechanisms and understand how EMS can be harnessed to fine‐tune cellular responses in tissue engineering applications. These insights open new avenues for therapeutic interventions targeting mechanosensitive ion channels, potentially assisting in recovery and rehabilitation processes following tendon injuries or related mechano‐pathologies.

For decades, mechano‐and electromagnetic wave therapies such as ultrasound or extracorporeal shock‐wave therapy have been explored in facilitating recovery and treating skeletal disorders post‐injury. This has led to an emerging trend in the use of whole‐body vibrating platforms as a strategy to sustain or boost musculoskeletal strength, particularly as part of aging management.^[^ ^83^, ^84^ ^]^ EMS differs fundamentally from these therapies in both its scale and precision of applied forces. Traditional therapies apply broad mechanical forces that may lack specificity and consistency in targeting cellular pathways. In contrast, EMS offers a highly controlled environment where both mechanical and electrical cues can be finely tuned at the nanoscale, allowing for precise modulation of cellular responses and providing a more effective approach to tendon tissue engineering and regenerative medicine applications. Overall, our findings add to the growing body of research demonstrating the role of bioelectricity in regulating cell differentiation.^[^ ^85^, ^86^, ^87^ ^]^

## 7 Conclusion 

Our study represents a significant step toward the development of more effective strategies for tissue engineering and regenerative medicine, particularly through the integration of bioelectric signals to control cell differentiation. This approach not only offers an alternative to genetic manipulation and expensive drug treatments but also opens new avenues for enhancing existing physical rehabilitation strategies. The ability to tune cellular responses by manipulating bioelectric signaling provides a more targeted and sustained therapeutic effect, which could lead to better outcomes in tissue repair and regeneration. Our study opens new avenues for the development of ion‐channel targeted biomaterials‐based therapies aimed at promoting tissue reinforcement and modulating mechano‐adaptation in mechanically inferior or diseased tendons, providing an alternative or complementary approach to existing physical rehabilitation strategies. Beyond tendon, our approach has broader implications for the regeneration of other mechanosensitive tissues, such as cartilage, bone, and even cardiovascular tissues

## 8 Limitations of the Study

The observed effects of EMS and MS on ion channel activity and downstream signaling pathways need to be validated in more extended culture periods and in vivo models to fully understand the long‐term implications of these stimuli on tendon regeneration. Additionally, the precise contribution of individual ion channels and the potential compensatory mechanisms that may occur when one pathway is inhibited were not fully explored. These limitations highlight the need for further research using more targeted genetic or pharmacological approaches to dissect the complex signaling networks involved.

## 9 Future Directions

Building on our findings, future research should focus on characterizing the specific signaling pathways activated by EMS and their interactions with mechanical stimuli. Advanced techniques such as CRISPR/Cas9 for gene editing or RNA interference for gene knockdown could be employed to investigate the roles of specific ion channels and signaling molecules in greater detail. Moreover, exploring the effects of EMS using biomimetic anisotropic fibrillar structures in combination with biochemical cues, such as growth factors or cytokines, could provide a more comprehensive understanding of how these different stimuli interact to influence tendon cell fate and function.

## 10 Experimental Section

### Film Fabrication by Spin Coating

A 12% polymer solution was prepared using a co‐solvent mixture of N,N‐dimethylacetamide (DMAc), and Acetone in a 6:4 ratio. This solution was then spin‐coated at a speed of 1000 rpm for 30 s, with an acceleration rate of 1 s to create a homogeneous film. Subsequent to each spin‐coating cycle, the films were baked at 50 °C. To fabricate thicker films, this process was reiterated thrice. Post the final spin‐coating, the pristine films were thermally annealed at 120 °C and composite films at 132 °C, each for 100 min to promote optimal crystallinity.

### BNNTs Dispersion

BNNTs (BNNT, LLC, USA), having a mass purity of 50% with traces of elemental boron, was used as it is without further purification or modification. For the experiment, a dose ranging from 0.5 to 10 mg of BNNTs was placed in a sample vial. A co‐solvent mixture (DMA:Acetone in a 6:4 ratio) was then added, resulting in a final concentration of 0.05–1 mg mL^−1^. The mixture was left to stir magnetically for a minimum of 4 days (96 h) to ensure adequate solvent‐nanotube interaction. Following this, the mixtures were sonicated for 3 h (10s ON, 2s OFF cycle) at an output power of 17 W. The homogenized dispersion was then centrifuged at 5000 rpm.

### Thermal Annealing

The annealing process was performed at 120 and 132 °C under vacuum for 2 h for pristine and composite films respectively, (between T_fusion_ and T_curie_) in a rapid thermal process. Following the annealing, a slow cooling method was employed to preserve the structural alterations. For this, the samples were allowed to cool to room temperature inside the oven, with the oven switched off. This gradual cooling aids in stabilizing the formation of the 𝛽‐phase within the film, which is particularly beneficial for enhancing the piezoelectric properties of the material.

### Thermoelectrical Poling

Thermoelectrical poling was performed to align the 𝛽‐phase dipoles. The process involved maintaining a maximum temperature of 70 °C to aid dipole rotation and alignment. To offset volume changes due to crystal rotations, the samples were subjected to compressive stress between electrodes. Finally, the samples were subjected to an electrical field greater than 50 MV m^−1^ for an hour to ensure effective dipole alignment, thereby enhancing the piezoelectric performance of the films.

### X‐Ray Diffraction (XRD)

XRD patterns were obtained (Philips X'pert PRO automatic diffractometer, 40 kV and 40 mA, with a theta–theta setup) to study the film's crystalline structures. The apparatus employed a Cu‐Kα radiation (λ = 1.5418 Å) and a PIXcel solid‐state detector. Data collection ranged from 5 to 70° 2θ, at room temperature. Fixed divergence and anti‐scattering slit were implemented for constant sample illumination. Signal deconvolution was assessed via WinPLOTR program's peak‐fit function. For profile fitting, pseudo‐Voigt functions were used, with a global FWHM and eta and a linear background. The Scherrer equation was used to deduce the crystalline domain's average size from the signal broadening.

### Thermal Transitions Analysis

The thermal behaviors of PVDF‐TrFE films were investigated through differential scanning calorimetry (DSC) and modulated differential scanning calorimetry (MDSC) methods. For this analysis, samples weighing 10 mg were sealed in aluminum pans. In the DSC analysis, a DSC Q200 from T.A. Instruments was utilized. The samples underwent heating from −10 to 200 °C at 10 °C min^−1^. The initial scan provided data on melting temperature (Tm), melting enthalpy (ΔHm), Curie temperature (Tc), and Curie enthalpy (ΔHc). The samples were then quenched within the DSC and subjected to a second scan under the same parameters. The non‐isothermal crystallization studies entailed heating the films to 260 °C at a constant rate of 30 °C min^−1^, maintaining this temperature for 2 min to erase thermal history, and then cooling the samples to room temperature under a nitrogen environment at the same rate. The heating protocol was similar to the isothermal crystallization kinetics, but cooling was interrupted at 148 °C and sustained for 10 min to ensure complete crystallization. The MDSC analyses were conducted using a TA Instrument 2000 MDSC. This allowed for an effective analysis of the microphase structures and molecular movements in the samples. An oscillation period of 60 s and a temperature amplitude of ± 0.47 °C were applied during the heating and cooling stages. Liquid nitrogen served as the cooling medium, with a purge rate of 50 mL min^−1^ for the samples. Each heating and cooling cycle commenced with a 3‐min isothermal hold for the samples. The overall process consisted of quenching the samples from room temperature to −75 °C, maintaining this temperature isothermally for 3 min, heating at a rate of 3 °C min^−1^ to 200 °C, and finally cooling at a rate of 3 °C min^−1^ down to 0 °C. The TA software for MDSC was employed for the recording, analysis, and deconvolution of the signals. All experiments were carried out as three replicates, with a standard deviation of less than 2%.

### Non‐Isothermal Crystallization Kinetics

The study of non‐isothermal crystallization kinetics was conducted employing the Avrami model and Jeziorny's modifications^[^ ^72^, ^73^, ^74^, ^75^ ^]^ Specifically, the degree of phase conversion was evaluated using the Avrami equation. Jeziorny's modification enabled crystallinity representation as a function of temperature under non‐isothermal conditions. Crystallization half‐time was computed utilizing the corrected kinetic constant derived from the Avrami equation. The Ozawa model was also used to dissect the nucleation and growth processes.

### Fourier Transform Infrared Spectroscopy (FTIR) Analysis

PVDF‐TrFE scaffolds were examined using a Nicolet AVATAR370 Fourier Transform Infrared Spectrophotometer operating in Attenuated Total Reflectance (ATR‐FTIR) mode. Spectra were collected at a resolution of 2 cm^−1^, averaged over 64 scans. The β‐phase fraction was identified and calculated based on the absorption bands associated with α and β phases at 532 and 846 cm^−1,^ respectively, using the previously described method.^[^ ^79^ ^]^ This method incorporates the absorbance values at 841 and 764 cm^−1^ and the ratio of absorption coefficients at these respective wavenumbers.

### Dielectric Properties

Dielectric characterization was carried out using a 2‐electrode configuration in an advanced electrochemical System (PARSTAT 2273). Samples were sputter‐coated with a 20 mm diameter gold as the electrode. A controlled A.C. electrical signal with an amplitude of 1 V rms was applied across a frequency range from 0.1 Hz to 1 MHz. The dielectric permittivity and the tangent loss components were extracted from the associated impedance amplitude and phase plots.

### Mechanical Properties

Tensile analysis of films was performed using Zwick/Roell Z010 with 1 kN load cell and a crosshead speed of 500 mm min^−1^ with a maximum extension of 500%. Films were prepared as per ASTM D 882 type specimen. Results are presented as average values with standard deviation (*r* = 5).

### Longitudinal Piezoelectric Measurement (d33 Coefficient)

The longitudinal piezoelectric constants were measured employing a d33 meter (PiezoTest, London, UK) with a precision level of 0.01 pC N^−1^ (pm V^−1^) for the longitudinal piezoelectric assessments. The samples with a load force of 10 N between two circular electrodes, each with an 18 mm diameter. Subsequently, a dynamic force of 0.25 N was exerted on the film's longitudinal axis at 110 Hz.

### Piezoresponse Force Microscopy (PFM)

The piezoelectric response of the samples was analyzed employing piezoresponse force microscopy (PFM, Bruker Nano Inc, Santa Barbara, CA, USA). For PFM assessments, a Pt–Ir coated conductive probe (SCM‐PIT) boasting a 2.8 N m^−1^ spring constant, and a 75 kHz resonant frequency was used. The amplitude of the piezoelectric signal detected was correlating with the material's piezoelectric coefficient (d33). Simultaneously, the signal's phase offers insights into the polarization direction of the domains. The average piezo‐response amplitude from ten locations was derived on a single sample. Standard PFM imaging verification was performed using the periodically poled lithium niobate (PPLN) test sample as a preliminary step. The vertical piezoresponse calibration was achieved through the AFM cantilever tip's deflection sensitivity, gleaned from the force‐displacement curve.

### Quantitative AFM Nano‐Mechanical Mapping

In PeakForce QNM, Young's modulus is calculated using a DMT model (see Equation 1) applied to the unloading portion of the force–separation curve. The DMT model can be viewed as a modified Hertzian model, which considers the adhesive forces between the tip and the surface. According to this approach, the reduced Young's modulus, Er, is given by:

(3) $$E_{r}=\frac{3\left(F_{tip}-F_{adh}\right)}{4\sqrt{Rd^{3}}}\;$$

In Equation 1, F_tip_ is the force on the AFM tip, F_adh_ is the adhesive force between the AFM tip and sample, R is the AFM tip radius, and d is the deformation depth. The reduced Young's modulus E_r_ is related to the sample Young's modulus Es:

(4) $$\frac{1}{E_{r}}=\frac{\left(1-v_{s}^{2}\right)}{E_{s}}+\frac{\left(1-v_{I}^{2}\right)}{E_{I}}$$

where E.I. is the indenter Young's modulus, vs. is the Poisson's ratio of the indenter, and $v_{s}$ is the Poisson's ratio of the sample. In the present work, E.I.>> E_s_, and so the second term on the right‐hand side of Equation 2 is negligible. The tip radius can be measured directly using a scanning electron microscope or a tip calibration grating. Alternatively, the value of the radius can be derived from a reference sample (in the present work, polystyrene) using Equation 1 and taking the modulus value to be determined using IIT. In this study, Poisson's ratio was assumed to be 0.49. AFM experiments were performed using a Bruker Dimension Icon AFM and two different probes: Bruker AFM Probes supplied PFTUNA and SCM‐PIT probes and were selected based on the recommendations for the range of polymer Young's moduli to be investigated (10–500 MPa). The experiments were carried out using an oscillation frequency of 2 kHz, and the amplitude was set to a constant value of 150 nm, corresponding to an indentation rate of 0.6 mm s^−1^. Before each experiment, the probes were calibrated, determining the tip radius and spring constant each time. Each set of Young's modulus measurements corresponds to 640 × 640 force‐separation curves obtained over an area of 15 × 15 µm.

### Vibration Apparatus

The bioreactor surface area was designed to hold two 6‐well plates (Corning, NY) simultaneously with dimensions of 130 × 178 mm. The piezo actuators used (PL088.30, Physik Instrumente, Karlsruhe, Germany) were low‐profile actuators with a large attachment area. The top plate of the platform used the magnetic attachment to secure the 6‐well plates. Standard 30 mm diameter, 3 mm thickness Fe_2_O_3_ ferrite magnets (Magnet Expert, Tuxford, UK) were bonded to the tissue culture plates. The quoted magnetic flux at the surface of these magnets was 700 gauss (0.07 T), which, as a static magnetic field, was not thought to be high enough to alter cellular function. However, these magnets, being Halbach arrays, were only magnetic on the side facing the bioreactor and away from the cell culture; therefore, any stray magnetic fields would be far smaller. To power the piezo array, a high‐voltage piezo driver (Model ENV 150, Piezosystem Jena, Germany) was used to provide 160 Vpk–pk. The sine wave modulation of the amplifier output was provided by a signal generator (Model 33 210 A, Agilent, Santa Clara, CA)

### Surface Functionalization for Enhanced Tendon Cell Proliferation

Chemical functionalization of the surface with relevant ligands is generally required for the prolonged culture of tendon cells on synthetic hydrophobic surfaces. Surfaces were treated with oxygen plasma for 45 s at 30 W and oxygen flow rate at 30 mL min^−1^. Briefly, radicals formed and reacted with oxygen to form hydroperoxides on the polymeric surface. Subsequently, hydroperoxides decomposed due to thermal gradients and produced secondary radicals that initiate the polymerization of acrylic acid (AAc). After oxygen plasma polymerization, treated surfaces were immersed in a 20% v/v AAc aqueous solution at 90 °C, which was previously purged with nitrogen using a reflux system to avoid changes in concentration. Next, the pAAc functionalized surfaces were rinsed with distilled H_2_0 for 18 h to remove unreacted monomers. Finally, carboxyl groups were activated with 0.1 m EDC and 0.1 m NHS (1:1) for 1 h immediately after the films were immersed in a fibronectin solution (10 µg mL^−1^) for 2 h at room temperature.

### Tenocytes and MSC Cell Culture

Human tendon‐derived cells were harvested from the patellar tendon during tendon grafting operations after obtaining written informed consent at the University Hospital Galway. From these tissue specimens, primary human tenocytes were isolated and cultured. They were identified as tenocytes through their characteristic growth pattern and detecting scleraxis (SCX) and tenomodulin (TNMD) expression. The studies were performed with cells at passages 2–3, and at least three donors were used for all assays. Primary bone marrow MSCs were sourced from Promocell (Heidelberg, Germany). All cells were maintained in Dulbecco's Modified Eagle's Medium (DMEM/F‐12 with Glutamax, Gibco‐BRL) supplemented with penicillin (100 U mL^−1^), streptomycin (10 µg mL^−1^) (both Sigma–Aldrich) and 10% fetal calf serum (Gibco‐BRL). Cells were detached by incubation with 0.05% trypsin for 5 min at 37 °C. l. All cell cultures were performed in an incubator at 37 °C with 5% CO_2_. The basal culture media was removed and replenished every 2–3 days.

### Cell Proliferation Assay

Tenocytes were seeded onto all experimental and control films in a modified 6‐well plate at 4.5 × 10^4^ cells/film (*n* = 3) for cell proliferation analysis. Alamar Blue assay (Sigma–Aldrich) assessed cell metabolic activity and proliferation. The cells were incubated in a medium supplemented with 10% (v/v) Alamar Blue dye for 4 h. A 100 µL sample of the medium with reduced Alamar blue was transferred, and the absorbance at 570 and 590 nm was measured in a 96‐well plate using a Varioskan Flash Plate reader. Non‐seeded biomaterial in the same medium was used as a negative control. The percentage of AlamarBlue reduction was calculated as follows:

(5) $$\begin{array}{cc} \mathrm{Reduction}\;\mathrm{of}\;\mathrm{Alamar}\;\mathrm{Blue}\;\left(\%\right) & =(\left(O2\;\times \;A1\right)-\left(O1\;\times \;A2\right)/\left(R1\;\times \;N2\right)\\ & \;-\left(R2\;\times \;N1\right))\;\times \;100 \end{array}$$

where O1 and O2 are the molar extinction coefficients of oxidized AlamarBlue at wavelengths 570  and 600 nm, respectively; R1 and R2 are the molar extinction coefficients of reduced AlamarBlue at wavelengths 570 and 600 nm, respectively; A1 and A2 are the observed absorbance readings for test wells at wavelengths 570 and 600 nm, respectively; and N1 and N2 are the observed absorbance readings for the negative control wells at wavelengths 570 and 600 nm, respectively.

### Live/Dead Assay

Viable cells were seeded at a density of 5000 cm^−2^ (*n* = 5) on PVDF films for quantitative analysis using fluorescent microscopy. Tenocytes were cultured for 1, 3, or 7 days. Untreated live and dead cells were used as controls for quantitative analysis. Live/Dead Assay (Life Technologies) visualized viable and necrotic cells. After each culture time, samples were washed with PBS and stained with calcein and ethidium bromide following the manufacturer's recommendations. The samples were immediately analyzed (after incubation) with a Varioskan Flash plate reader. Samples were subsequently imaged on an Olympus IX81 inverted fluorescent microscopy with the 20× objective.

### Protein Extraction

Cells were scraped and washed with ice‐cold PBS containing calcium and magnesium to preserve cellular integrity and ensure effective lysis. The cells were then lysed in ice‐cold radioimmunoprecipitation assay (RIPA) buffer [1% Triton X‐100, 1% sodium deoxycholate, 0.1% SDS, 150 mm NaCl, 50 mm Tris‐HCl (pH 7.4)] supplemented with Protease Inhibitor Cocktail (ThermoFisher, diluted 1:100) and Phosphatase Inhibitor Cocktail (ThermoFisher, diluted 1:100) to prevent protein degradation and dephosphorylation. The samples were subjected to brief sonication (3 cycles of 5 s on/15 s off at 4 °C, at 20% amplitude) to shear DNA and reduce sample viscosity, preventing interference with subsequent steps. Lysis was allowed to proceed on ice for 20 min to ensure complete disruption of cellular membranes. The lysates were then clarified by centrifugation at 14 000 × g for 15 min at 4 °C to remove insoluble debris. Protein concentration was determined using a Bicinchoninic Acid (BCA) protein assay kit (Pierce), following the manufacturer's instructions, with bovine serum albumin (BSA) as a standard. Samples were diluted as necessary to ensure that they fell within the linear range of the assay.

### Protein Microarray

The Protein Antibody Microarray was custom‐made. Nexterion slide H microarray slides were purchased from Schott AG (Mainz, Germany). Alexa Fluor 555 carboxylic acid succinimidyl ester was obtained from Life Technologies (Carlsbad, CA, USA). According to the manufacturer's instructions, protein samples were labeled with Alexa Fluor 555 carboxylic acid succinimidyl ester. The excess label was removed, and the buffer was exchanged with PBS, pH 7.4, by centrifugation through 3 kDa molecular weight cutoff filters. Absorbance at 555 and 280 nm was measured for labeled samples, and calculations were performed according to the manufacturer's instructions using an arbitrary extinction coefficient of 100 000 and molecular mass of 100 000 to enable quantification of relative protein concentration and label substitution efficiency. All commercial antibodies were buffer exchanged into PBS and quantified by bicinchoninic acid (BCA) assay. Antibodies were diluted to print concentration in PBS and printed in six replicates on Nexterion H amine‐reactive, hydrogel‐coated glass slides using a SciFLEXARRAYER S3 piezoelectric printer (Scienion, Berlin, Germany) under constant humidity (62% +/− 2%) at 20 °C. Each feature was printed using ≈1 nL of diluted antibody using an uncoated 90 µm glass nozzle with eight replicated subarrays per microarray slide. After printing, slides were incubated in a humidity chamber overnight at room temperature to facilitate complete conjugation. The slides were then blocked in 100 × 10^−3^ m ethanolamine in 50 × 10^−3^ m sodium borate, pH 8.0, for 1 h at room temperature. Slides were washed in PBS with 0.05% Tween 20 (PBS‐T) three times for 2 min each wash, followed by one wash in PBS, dried by centrifugation (470 × g, 5 min), and then stored with a desiccant at 4 °C until use. Incubations were carried out in the dark. Microarray slides were incubated as previously described. Initially, one labeled sample was titrated (2.5–15 µg mL^−1^) for optimal signal‐to‐noise ratio, and all samples were subsequently incubated for 1 h at 23 °C at 9 µg mL−1 in Tris‐buffered saline (TBS; 20 × 10^−3^ m Tris‐HCl, 100 × 10^−3^ m NaCl, 1 × 10^−3^ m CaCl2, 1 × 10^−3^ m MgCl_2_, pH 7.2) with 0.05% Tween 20 (TBS‐T). All microarray experiments were carried out using three replicate slides. Alexa Fluor 555 labeled cells lysate (10 µg mL^−1^) were incubated in two separate subarrays on every slide to confirm retained antibody performance and printing. After incubation, slides were washed three times in TBS‐T for 2 min per wash, once in TBS, and then centrifuged dry as above. Dried slides were scanned immediately on an Agilent G2505 microarray scanner using the Cy3 channel (532 nm excitation, 90% photomultiplier tubes (PMT), 5 µm resolution), and intensity data were saved as a .tif file. Antibody microarrays were verified to remain active for at least 2 weeks after printing, and all incubations were carried out within that timeframe. Data extraction from .tif files was performed mainly as previously described. Data were normalized to the mean of three replicate microarray slides (subarray‐by‐subarray using subarray total intensity, *n* = 4, 24 data points). Unsupervised hierarchical clustering of normalized data was performed using Hierarchical Clustering Explorer v3.0 (http://www.cs.umd.edu/hcil/hce/hce3.html) using the parameters no prefiltering, complete linkage, and Euclidean distance. All data presented here were confirmed using at least four replicates for each test and control group. The results are expressed as the mean of the values ± standard error of the mean.

Different types of arrays were fabricated to investigate tendon regeneration or phenotype maintenance (tenogenesis, **Table** **2**), intracellular molecular pathways (signaling, **Table** **3**), or membrane proteins (receptors, **Table** **4**). The regulation of osteogenic markers BMP was assessed by measuring Phosphorylated Smad1/5/8 (BMP Pathway, Cell Signaling, 5753S) relative to pan control (Total Smad1 and Smad5, Cell Signaling 6944S and 12534S). The activation of the WNT osteopecific was assessed by measuring active β‐catenin (clone 8E7 (Millipore, ABC) relative to pan control (Total β‐Catenin (WNT Pathway Markers, Millipore, 2858901).

**Table 2:** Tenogenesis array. Proteins associated with tendon regeneration or tenocyte function.

Proteins	Abbreviation	Purchased from
Scleraxis	SCX	Abcam
Tenomodulin	TNMD	Abcam
Byglican	BGN	Abcam
Decorin	DCN	Abcam
Thrombospondin 4	THBS‐4	Abcam
Tenascin C	TNC	Abcam
Collagen I	COLI	Abcam
Collagen III	COLIII	Abcam
Collagen V	COLV	Abcam

**Table 3:** Signaling array. Proteins are associated with different signaling pathways (MAPK, FAK, TGF‐B, BMP, and WNT).

Proteins	Abbreviation	Purchased from
Smad 1	SMAD1	Cell signaling
Smad 3	SMAD3	Cell signaling
Smad1/5/8	SMAD1/5/8	Cell signaling
Phosphorylated Smad 1/5/8	pSMAD158	Cell signaling
Focal adhesion kinase	FAK	Cell signaling
Phosphorylated FAK	pFAK	Cell signaling
MAPK	ERK1/2	Cell signaling
pMAPK	pERK1/2	Cell signaling
Wnt/ β‐catenin	β ‐Catenin	Millipore
Active β‐catenin	Active β‐Catenin	Millipore

**Table 4:** Receptors array. Proteins associated with cell membrane receptors.

Proteins	Abbreviation	Purchased from
TRPV1	TRPV1	Abcam
Piezo1	Piezo1	Abcam
Piezo2	Piezo2	Abcam
TRPA1	TRPA1	Abcam
KCNK2	KCNK2	Abcam
KCNK4	KCNK4	Abcam
L‐type Ca^2+^	L‐type Ca^2+^	Abcam
BMPR1A	BMPR1A	Abcam
Integrin1	ITG1	Abcam
Integrin3	ITG3	Abcam
Integrin5	ITG5	Abcam

### RT2‐qPCR Array Processing

Total RNA was extracted from samples using a Trizol reagent followed by chloroform precipitation, and further purified using a Qiagen RNeasy column to ensure high purity. The quality and integrity of the purified RNA were assessed using an Agilent 2100 Bioanalyzer in conjunction with the RNA 6000 Pico Kit, particularly for low sample quantities (Agilent Technologies, USA). The RNA Integrity Number (RIN) and RNA concentration were determined, with only samples showing RNA concentrations above 20 ng µL^−1^ and RIN values greater than 9 being selected for subsequent cDNA synthesis. cDNA synthesis was performed according to the manufacturer's protocol using the RT2 First Strand Kit (SA Biosciences). This involved the reverse transcription of RNA into cDNA. The PCR amplification was conducted using an iQ5 Thermal Cycler (Bio‐Rad, Munich, Germany). The reaction setup included 10 µL of synthesized cDNA, 10 µL of Genomic DNA Elimination Mixture, and 91 µL of RNase‐free water, combined with the RT2 SYBR Green qPCR Master Mix (SA Biosciences), following the manufacturer's guidelines. Each experiment was performed in triplicate (*N* = 3), with technical duplicates for each gene set analyzed to ensure reproducibility. Quantification of gene expression was normalized against a panel of stable housekeeping genes, including TOP1, GAPDH, HSP90AB1, RPLP0, and TBP. The normalization was conducted using the geometric mean of the threshold cycle (Ct) values of these reference genes on each plate. Relative expression changes were calculated using the ΔΔCt method, which was then used to determine fold changes in gene regulation (**Table** **5**).

**Table 5:** QIAGEN genes list for customized gene array.

Symbol	Entrez gene name
ABCB1	ATP binding cassette subfamily B member 1
ACAN	Aggrecan
ACTA1	actin, alpha 1, skeletal muscle
ACVR1	activin A receptor type 1
AHSG	alpha 2‐HS glycoprotein
ALPL	alkaline phosphatase, liver/bone/kidney
BGLAP	bone gamma‐carboxyglutamate protein
BGN	Biglycan
BMP1	bone morphogenetic protein 1
BMP2	bone morphogenetic protein 2
BMP4	bone morphogenetic protein 4
BMP6	bone morphogenetic protein 6
BMP7	bone morphogenetic protein 7
BMPR1A	bone morphogenetic protein receptor type 1A
BMPR2	bone morphogenetic protein receptor type 2
CASP3	caspase 3
COL11A1	collagen type XI alpha 1 chain
COL14A1	collagen type XIV alpha 1 chain
COL1A1	collagen type I alpha 1 chain
COL1A2	collagen type I alpha 2 chain
COL2A1	collagen type II alpha 1 chain
COL3A1	collagen type III alpha 1 chain
COL4A1	collagen type IV alpha 1 chain
COL5A1	collagen type V alpha 1 chain
COL6A1	collagen type VI alpha 1 chain
COMP	cartilage oligomeric matrix protein
DCN	Decorin
DLX5	distal‐less homeobox 5
EGR1	early growth response 1
FGF10	fibroblast growth factor 10
GDF15	growth differentiation factor 15
GDF5	growth differentiation factor 5
GDF6	growth differentiation factor 6
GDF7	growth differentiation factor 7
HAT1	histone acetyltransferase 1
HDAC1	histone deacetylase 1
HNF1A	HNF1 homeobox A
IBSP	integrin binding sialoprotein
IGF1	insulin like growth factor 1
ITGA1	integrin subunit alpha 1
ITGA2	integrin subunit alpha 2
ITGA3	integrin subunit alpha 3
ITGA4	integrin subunit alpha 4
ITGA5	integrin subunit alpha 5
ITGAX	integrin subunit alpha X
ITGB1	integrin subunit beta 1
ITGB3	integrin subunit beta 3
ITGB5	integrin subunit beta 5
KAT2B	lysine acetyltransferase 2B
KCNK2	potassium two pore domain channel subfamily K member 2
KCNK4	potassium two pore domain channel subfamily K member 4
KDR	kinase insert domain receptor
MGP	matrix Gla protein
MKX	mohawk homeobox
PIEZO1	piezo type mechanosensitive ion channel component 1
PIEZO2	piezo type mechanosensitive ion channel component 2
PIK3CG	phosphatidylinositol‐4,5‐bisphosphate 3‐kinase catalytic subunit gamma
PTEN	phosphatase and tensin homolog
PTK2	protein tyrosine kinase 2
PTK2	protein tyrosine kinase 2
PXN	Paxillin
RUNX2	runt related transcription factor 2
SCX	scleraxis bHLH transcription factor
SMAD3	SMAD family member 3
SMAD4	SMAD family member 4
SMAD9	SMAD family member 9
SMURF1	SMAD specific E3 ubiquitin protein ligase 1
SMURF2	SMAD specific E3 ubiquitin protein ligase 2
SOX9	SRY‐box 9
SP7	Sp7 transcription factor
SPARC	secreted protein acidic and cysteine rich
SPP1	secreted phosphoprotein 1
TBX5	T‐box 5
TGFB1	transforming growth factor beta 1
THBS4	thrombospondin 4
TLN1	talin 1
TNC	tenascin C
TNMD	Tenomodulin
TRPA1	transient receptor potential cation channel subfamily A member 1
TRPV1	transient receptor potential cation channel subfamily V member 1
TWIST1	twist family bHLH transcription factor 1
VCL	Vinculin
VEGFA	vascular endothelial growth factor A
ZYX	Zyxin

### Mathematical Model of Shear Stress Generated by Vibrating Diaphragm

Shear Stress (τ) near the vibrating film can be approximated by:

(6) τ≈η·A·ω·k

where A is the amplitude of the vibration (assumed to be ≈100 nm), ω is the angular frequency, and k is the wavenumber.

Angular Frequency (ω) is calculated as ω = 2π × f, where f is the frequency of the vibration (1500 Hz).

Wavenumber (k) is calculated as k = √(ωρ)/2η, where ρ is the density of the fluid (≈1000 kg m^−^^3^ for water), and η is the dynamic viscosity of the fluid (≈0.001 Pa s for cell culture media).

Calculated Values: A = 500 nm, ω ≈ 9424 rad s^−1^, k ≈ 6.86 × 10^4^ m^−1^, τ ≈ 0.32 Pa

### Cytoskeletal Inhibition

T.C.s were seeded at 7.5 × 10^3^ cells cm^−2^ in stimulated and control culture conditions for 5 days in the presence or absence of inhibitors of ROCK (Y27632, Sigma) at 10 µm and myosin II (blebbistatin, Sigma cat. B0560) at 5 um. Inhibitors were added fresh with all medium changes for the duration of the experiment.

### Statistical Analysis

All statistical analyses were conducted using Minitab 11. Data were pre‐processed by normalizing across samples to account for variability and ensure consistency in comparisons. Outliers were identified and evaluated using the Grubbs test, and any significant outliers were excluded from further analysis. Data are presented as mean ± standard deviation (SD) unless otherwise specified.

Sample sizes (n) for each experiment are indicated in the figure captions and were determined based on preliminary power analysis to ensure adequate statistical power. For single comparisons, a two‐tailed Student's t‐test was used to determine significant differences between groups. For comparisons involving more than two groups, one‐way ANOVA was applied, followed by Tukey's post hoc test. The level of significance (alpha) was set at 0.05 for all tests. Assumptions for each statistical test, including normality and homogeneity of variances, were checked using the Shapiro–Wilk and Levene's tests. All relevant figure captions include detailed information on the sample size (n), the probability (P) value, the specific statistical test used, the presentation of data, and the meaning of significance symbols used in the figures.

## Conflict of Interest

The authors declare no conflict of interest.

## Supporting information



Supporting Information

## Data Availability

The data that support the findings of this study are available in the supplementary material of this article.
