# Supplementary material for: A Tympanic Piezo‐Bioreactor Modulates Ion Channel‐Associated Mechanosignaling to Stabilize Phenotype and Promote Tenogenesis in Human Tendon‐Derived Cells
Source: Adv Sci (Weinh). 2024 Oct 22;11(45):2405711. doi: 10.1002/advs.202405711 (PMC11615817; doi:10.1002/advs.202405711)
Supplement: Supplementary file 1 — Supporting Information [file ADVS-11-2405711-s001.docx]

# Supporting Information

a


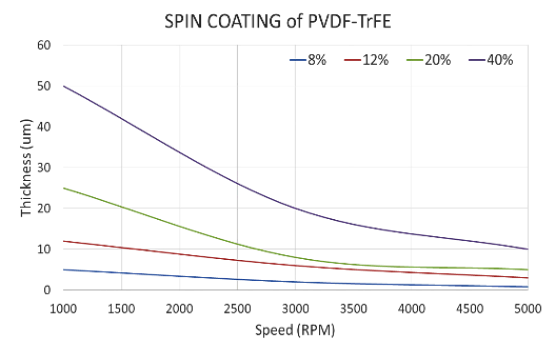


b
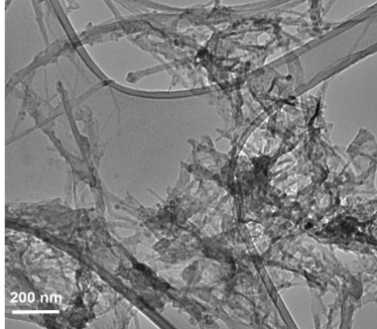
 c
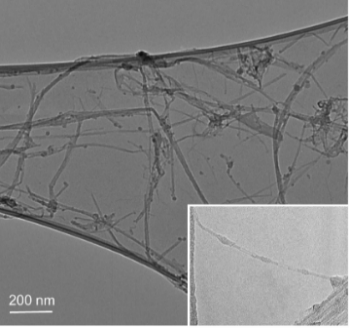
 d
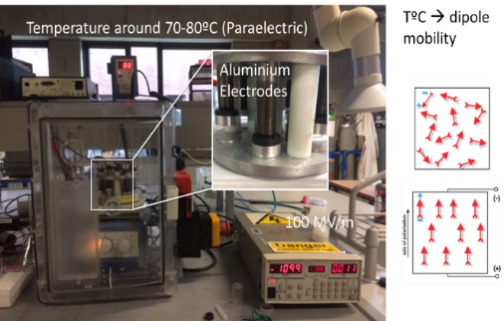


**Figure S1.** a) Resultant thickness spin coating of pVDF-TrFE with different concentrations. b) Strong aggregation in BNNT as a consequence of the polarity of B-N bond, which leads to more complicated intertube interactions c) Disaggregated nanotubes after functionalization d) Custom-made thermal electrical poling rig.

b

**c**

**Figure S2**. MDSC of PVDF-TrFE (a) as received, (b) annealed at 120 ˚C and (c) PVDF-TrFE/BNNT heating curves heating and cooling cycles.


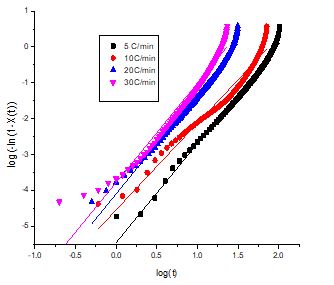


Composite

Annealed

Pristine


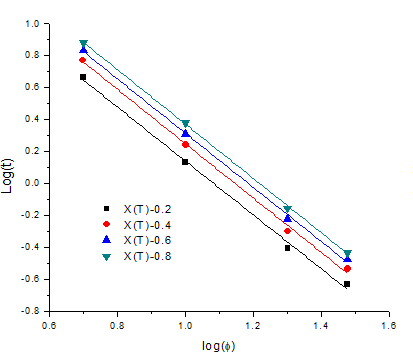


Annealed

Pristine

Composite


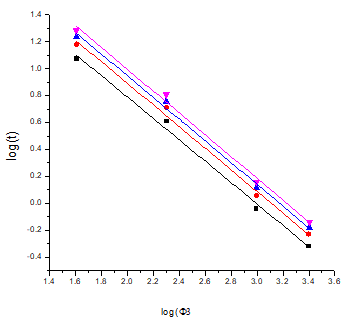


Annealed

Pristine

Composite

**Figure S3.** a) Avrami plot, b)1st and c) 2nd peak of PVDF TrFE pristine and annealed. The non-isothermal crystallization analysis is based on the primary crystallization stage.

At the early stage of crystallization, ln[-ln(1 - Xt)] is in good linear relation with lnt (R2 close to 1); therefore, the modified Avrami equation is suitable for these systems. The Avrami exponent n of neat PVDF-TrFE decreases with increasing cooling rate and is mainly associated with crystallization growth. The n values of PVDF-TrFE (n=3,55 ± 0,19) indicate that crystal growth of pristine and annealed PVDF-TrFE should be a spherical three-dimensional process. The highest values are obtained for PVDF-TrFE annealed (n= 4,00 ± 0,19), forming a more significant number of crystals. This suggests that annealing results in more nucleation sites, whereas pristine offers larger crystallization growth.

Similarly, the value t1 is also associated with the crystallization rate; the smaller this value is, the faster the crystallization process. Although higher n values are generally associated with the formation of smaller crystals (heterogeneous crystallization), crystal formation in annealed PVDF-TrFE showed a slightly higher growth time (t1/2), suggesting a slower crystallization rate and bigger crystals compared to pristine PVDF-TrFE. This was observed in XRD analysis, where the crystallite size increased after annealing.

**Table S1**. Avrami 1^st^ peak parameters of PVDF TrFE pristine and annealed. First crystals (Higher temperature crystals).

| Sample Type | $\phi$  ( ̊ C/min) | n | Log k | k’ | t_1/2_  (Sec.) | R^2^ |
| --- | --- | --- | --- | --- | --- | --- |
| Pristine | 5 | 3.75 | -6.3 | 5.0119E-07 | 43.40 | 0.998 |
|  | 10 | 3.67 | -5.2 | 6.3096E-06 | 23.63 | 0.999 |
|  | 20 | 3.41 | -4.0 | 8.7096E-05 | 13.92 | 0.999 |
|  | 30 | 3.37 | -3.4 | 0.00039811 | 9.15 | 0.999 |
| Annealed | 5 | 3.71 | -6.4 | 3.9811E-07 | 48.10 | 0.998 |
|  | 10 | 4.11 | -6.2 | 5.8884E-07 | 29.99 | 0.995 |
|  | 20 | 3.99 | -5.2 | 6.3096E-06 | 18.33 | 0.995 |
|  | 30 | 4.00 | -4.7 | 1.9498E-05 | 12.64 | 0.995 |
| Composite | 5 | 3.01 | -4.66 | 2.16E-05 | 31.4 | 0.990 |
|  | 10 | 3.01 | -3.98 | 0.000104 | 18.62 | 0.985 |
|  | 20 | 3.18 | -3.44 | 0.000363 | 10.75 | 0.995 |
|  | 30 | 3.01 | -3.09 | 0.000813 | 9.41 | 0.975 |

**Table S2**. Avrami 2^nd^ peak parameters of PVDF TrFE pristine and annealed. Second crystals (Lower temperature crystals)

| Sample Type | $\phi$  ( ̊ C/min) | n | Log k | k’ | t_1/2_  (sec) | R^2^ |
| --- | --- | --- | --- | --- | --- | --- |
| Pristine | 5 | 2.75 | -4.6 | 1.5849E-06 | 49.42 | 0.998 |
|  | 10 | 2.83 | -4.3 | 6.3096E-07 | 42.91 | 0.996 |
|  | 20 | 2.90 | -3.4 | 7.9433E-06 | 25.09 | 0.996 |
|  | 30 | 3.01 | -3.6 | 6.3096E-05 | 13.25 | 0.994 |
| Annealed | 5 | 2.67 | -4.7 | 2.0417E-05 | 44.41 | 0.998 |
|  | 10 | 2.89 | -4.4 | 4.5709E-05 | 30.01 | 0.995 |
|  | 20 | 2.83 | -3.5 | 0.00039811 | 13.10 | 0.995 |
|  | 30 | 2.94 | -3.3 | 0.00022909 | 14.33 | 0.995 |
| Composite | *5* | 2.75 | -5.44 | 3.55E-06 | 83.91 | 0.987 |
|  | *10* | 2.42 | -4.53 | 2.88E-05 | 64.59 | 0.958 |
|  | *20* | 2.79 | -4.09 | 8.08E-05 | 25.69 | 0.979 |
|  | *30* | 2.77 | -3.77 | 0.000168 | 20.19 | 0.969 |

a b c

d e f


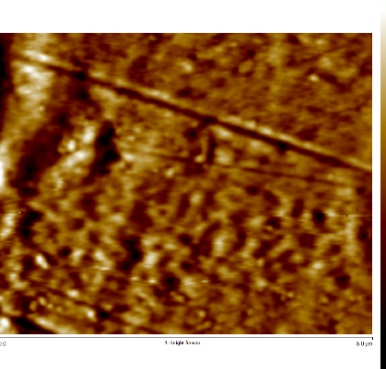

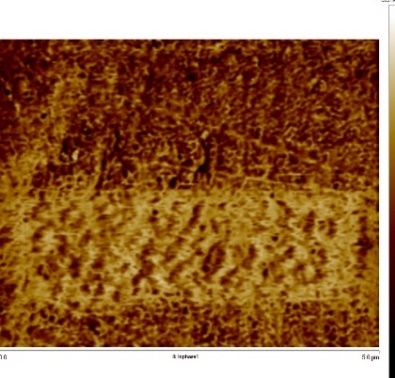

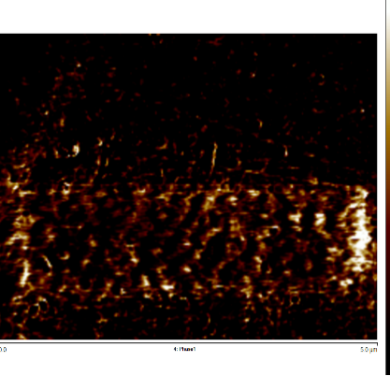


g h


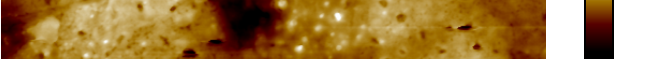

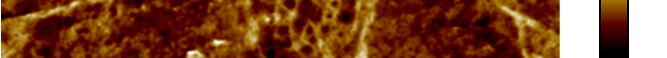


**Figure S4.** (a)Tensile tests of PVDF-TrFE and PVDF-TrFE-BNNT 1 wt. % composites at room temperature b) Electrical properties of PVDF-TrFE pristine and c) PVDF-TrFE/BNNT composite thin-film. AFM Images of a pristine PVDF-TrFE after poling by +/−20V d) Topography e) Piezoresponse Amplitude f) Piezoresponse g) Phase Topography and h) amplitude channels of composite samples.

In dynamic conditions, the system is exposed to vibrations that generate stress-strain fields in the material. In turn, the stress-strain field is translated into electrical charges at the surface of the piezoelectric material. Therefore, the system can be electrically modeled as a current source that charges a “capacitor” (formed by the upper and bottom surfaces of the piezoelectric film). In comparaison, the system is vibrating close to the resonance model, which can be represented by a current source *IP* connected in parallel with a capacitor *(C_P_)*  and a resistor (*R_P_)*. As the vibration follows a sinusoidal wave, the current source can be described by *I(t)* = *I*0 sin2*πf*0*t*, where *f*0 is the excitation frequency. The theoretical electric power generated by a piezoelectric harvester is given as:

$V(t)=I(t).Z_{INT}$ (2)

where  *I*  is the amplitude of the current source and *Zint* is the equivalent internal impedance of the system. Our system consists of a circular membrane clamped at the periphery and free to oscillate, also called a diaphragm. When observing the boundary conditions, we see a heterogeneous distribution of strain in the piezoelectric layer while vibrating, the volumetric strain is higher at the middle of the membrane and near the clamped end and very little between these two areas (see Figure SI5).


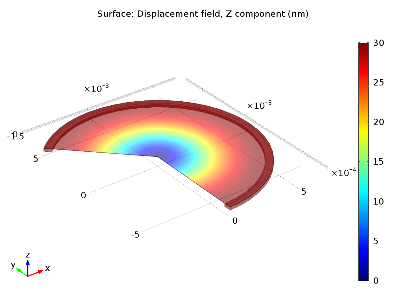

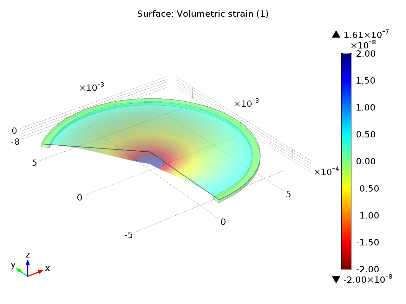

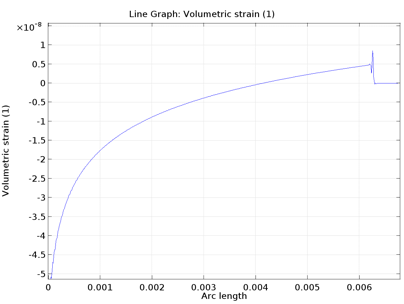


**Figure S5.** 3D Membrane displacement, 3D membrane volumetric strain and volumetric strain vs radius

a b

 ****

**Figure S6**. a) Membrane resonance frequency as a function of change in applied tension. b) Interferometric calibration of the bioreactor for one frequency (200 Hz). Vibration amplitude and output voltage are linearly dependent on the input voltage/displacement of the platform.

The media surrounding a vibrating membrane dramatically affects the structure, adding “virtually” mass and damping. Generally, it is known that the natural frequencies of structures interacting or fully immersed with water decrease significantly (compared to in air). The membrane’s vibration is transferred to the water movement and increases the total kinetic energy of the composite system. If the mode shapes do not change under the influence of water, the following approximate formula can be used to predict the natural frequency change when the circular membrane is in contact with water:

$f_{w}=f_{a}/\sqrt{1+\beta.\Gamma}$ (3)

Where β is a thickness correction factor and Γ is the non-dimensional added virtual mass incremental (NAVMI) factor. The main properties of the liquid that impact the resonant frequency are viscosity, density and pressure. Viscosity has a negligible impact on resonant frequency for fluids with viscosities less than 0.006 N⋅s/m2 (water viscosity is 0.001 N⋅s/m2 and air viscosity is 0.000018 N⋅s/m2). The viscosity dissipates system energy resulting in a faster decay in free vibration and reducing the resonant frequency amplitude at forced vibration. Inaba et al. showed that the resonant frequency decreased with higher viscosity. However, no shift was observed for the frequency of the resonance^35^. Physically, a shift in resonant frequency is attributed exclusively to an added mass effect. Briefly, the movement of the fluid in phase with the relative movement of the vibrating membrane results in a virtually added mass to the membrane. Lamb et al. first described this effect, which showed that membrane-fluid interactions could be described by the ratio between the kinetic energies of the membrane and fluid, a factor known as an added virtual mass incremental factor, or AVMI factor. The AVMI method can be used to predict the effect of fluid loading on vibrating structures. Kwak and Kim showed how the AVMI factor could be used to accurately predict the resonant frequency of a circular membrane under various clamping conditions^36^. They showed that the AVMI factor, β, could be solved merely as:

$\beta.\Gamma=\frac{\rho_{f}}{\rho_{p}}.\frac{r_{p}}{t_{p}}$ (4)

with: ρ_f_: fluid density ρ_P_: membrane density r_P_: plate radius t_p_: plate thickness Γ: non-dimensional added virtual mass incremental (AVMI) factor accounting for vibration mode and clamping.

**Table S3.** Non-dimensional added virtual mass incremental factor Γ for a uniform thickness circular membrane. Values of NAVMI factors for a uniform thickness circular plate. In this table, ‘*n*’ represents the order of the mode number, and ‘*N*’ denotes the number of terms used in the interpolation function to approximate the mode shape.

| Modes | *n* | | | | | | | |
| --- | --- | --- | --- | --- | --- | --- | --- | --- |
|  | 0 | 1 | 2 | 3 | 4 | 5 | 6 |  |
|  | 0,6669 | 0,2807 | 0,1671 | 0,1182 | 0,082 | 0,0651 | 0,0541 |  |

The solution becomes extremely difficult because although the membrane solution is two-dimensional, the fluid movement is three-dimensional, adding complexity. It is important to note that the first two orders of modal NAVMI factors are far bigger than the NAVMI factors. That indicates that the first two vibration modes are more sensitive to water than the other modes.

**Table S4**. Parameters for calculating the thickness factor

| Plate radius, r_p_ | Plate thickness, t_p_ | Membrane density, ρ_p_ | Water density, ρ_w_ |
| --- | --- | --- | --- |
| 6,25 mm | 25 µm | 1,78 x 10^3^ kg/m^3^ | 1,0 x 10^3^ kg/m^3^ |

In this study, the following parameters for the uniform circular plate are found in table SI4 and the value for β.Γ is 140,5. Taking the AVMI factor for the first natural frequency then we obtain the following:

$\frac{f_{w}}{f_{a}}=$0,103 for *n=1and fw =689,9 Hz*

**Table S5**. Obtained and calculated results of the first resonant frequencies.

|  |  | AIR |  | WATER | | |
| --- | --- | --- | --- | --- | --- | --- |
| Modes | Analytical | COMSOL | Observation | Analytical | COMSOL | Observation |
| 1 | 3270,8 | 2833,9 | 3800 | 689,94 | 696,1 | 700 |
| 72 | 4384,2 | 4250,9 | - | 1232,5 | 1500,2 | 1500 |
| 3 | 4619,7 | 6778,1 | - | 1325,3 | 2500,3 | 2500 |

It is also observed that the natural frequencies of the membrane exposed to water are smaller than those exposed to air, and the natural frequencies of the membrane exposed to water on both sides are smaller than those of the circular plate exposed to water with one side.

| AIR  a | WATER |
| --- | --- |
|  |  |
| b | |
|  |  |
| c | |
|  |  |

**Figure S7.** The effect of membrane-fluid interactions results in the shift of resonant frequency.(a) PVDF-TrFE Poled (b) Nanocomposite and (c)PVDF_TrFE non-poled

The hole size (membrane diameter) affected the voltage output and therefore, the sensitivity. As observed, when the hole was smaller than 6 mm, the sensitivity was lower than 0,1 mV/nm. The sensitivity increases with hole-size up to 12.5 mm. Further increasing the diameter resulted to increase in the sensitivity.

|  |  |
| --- | --- |
| a   | b |
| **Figure S8**. Sensitivity of the stimulator devices. Effect of (a) hole diameter (membrane thickness, 25 µm; sound wave frequency, 158)0 Hz; displacement 30 nm), the (b) thickness of the membrane (hole diameter, 12.5 mm; vibrational wave frequency, 1500 Hz; 30 nm displacement). | |

a


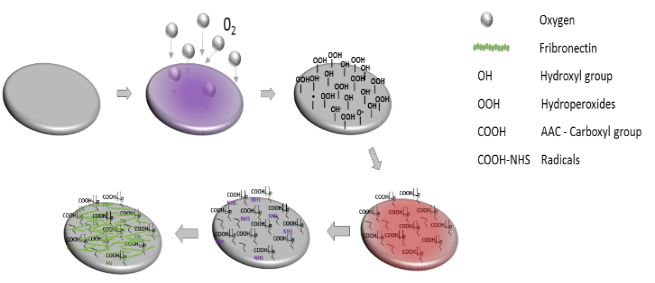

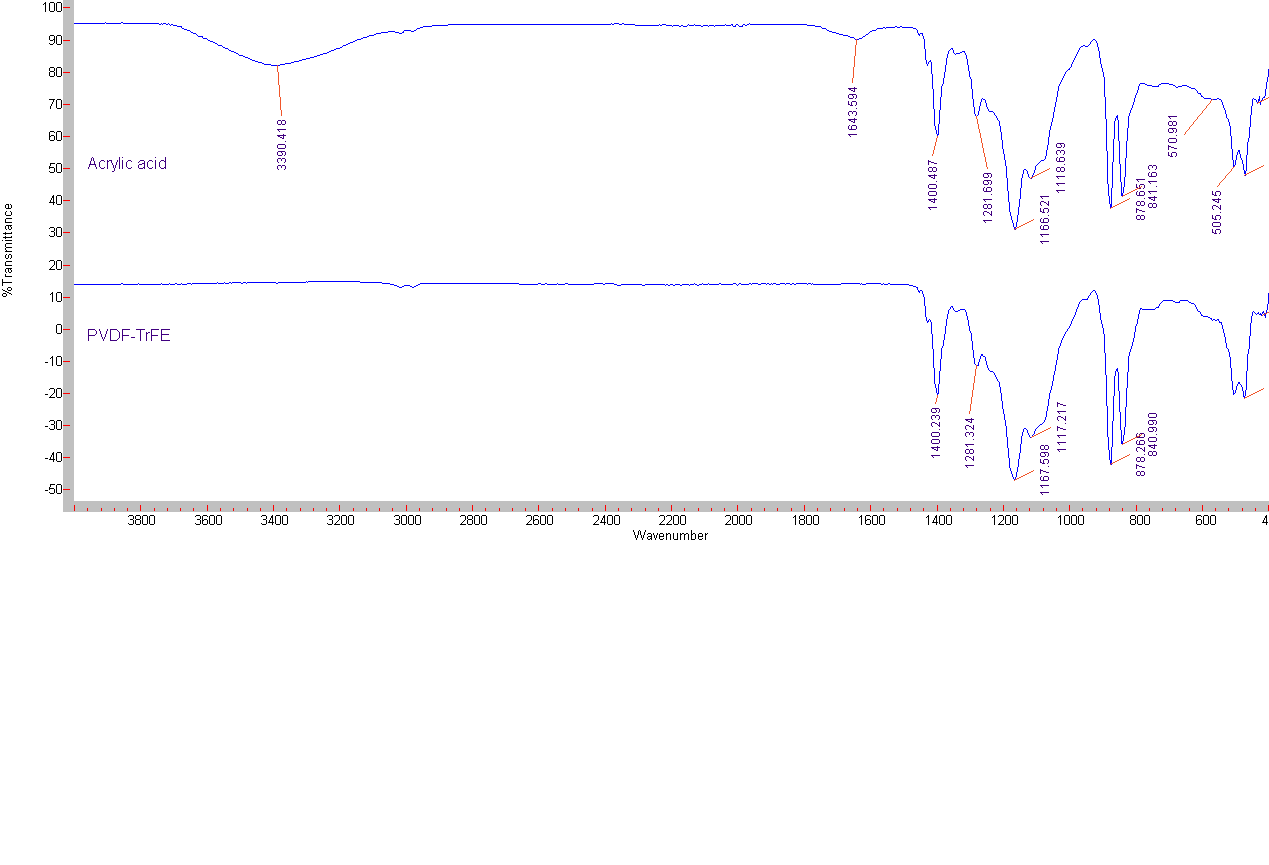


b


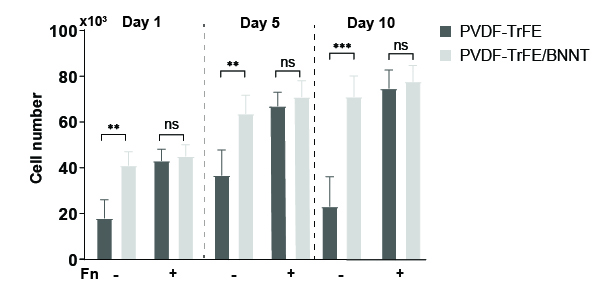


**Figure S9**. Surface modification method using oxygen (low temperature) plasma treatment of the PVDF-TrFE surface. The bombardment of the surface with oxygen ions results in the formation of hydroperoxides that can be used to graft poly acrylic acid on the surface and maximize the surface energy to allow fibronectin attachment through carbodiimide chemistry. FTIR shows functional groups after polyacrylic acid reaction on the PVDF-TrFE surface.b) Proliferation behaviour of human tenocytes cells on PVDF-TrFE and nanocomposite PVDF-TrFE/BNNT substrates for 1, 5, and 10 days with and without Fn covelant coating. Graphs show mean and standard deviation statistics by one-way ANOVA with multiple comparisons, n ≥ 15: *, p < 0.05; **, p < 0.01; ***, p < 0.001; ****, p < 0.0001; n = 3 biological donor.


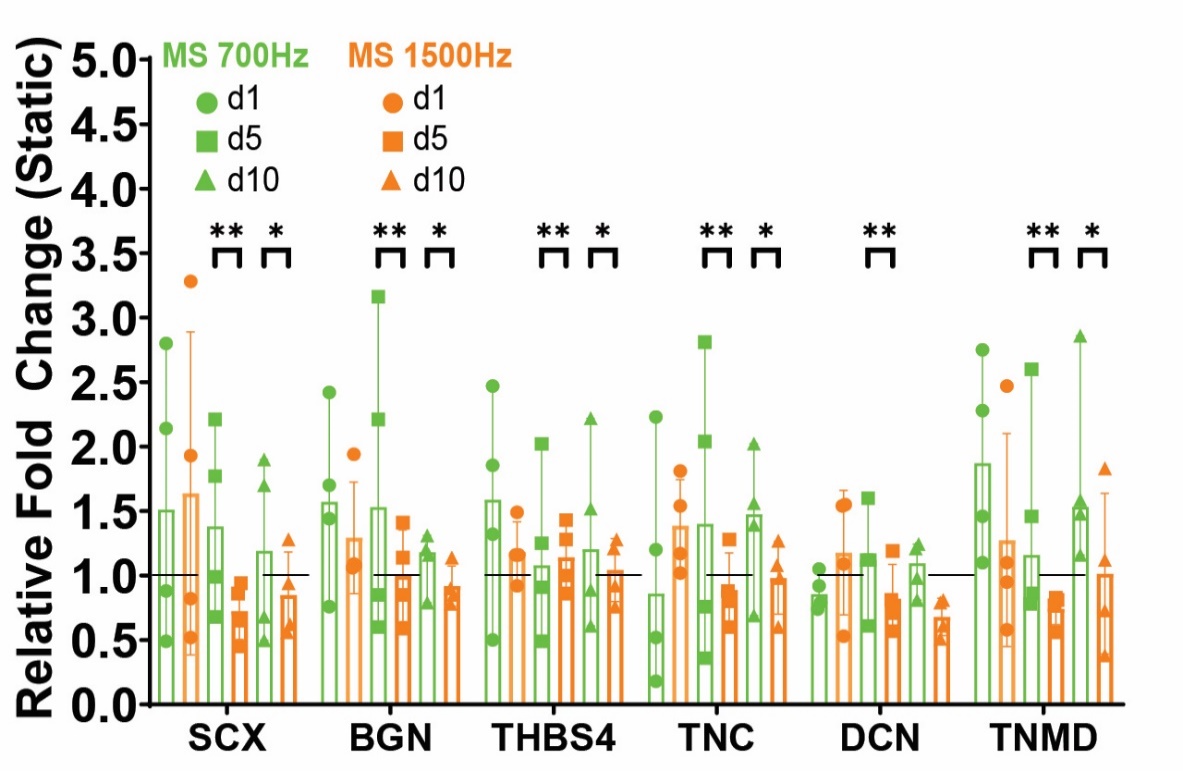


**Figure S10**. Expression of proteins related to tenogenesis on hTDCs under Mechanical stimulation using non-piezoelectric films at (a) 700 Hz and (b) 1500 Hz for 1,5 and 10 days. Data is presented as Median ± IQR (N=4). (***p<0.001, **p<0.01 ; *p<0.05; to respect to static control).


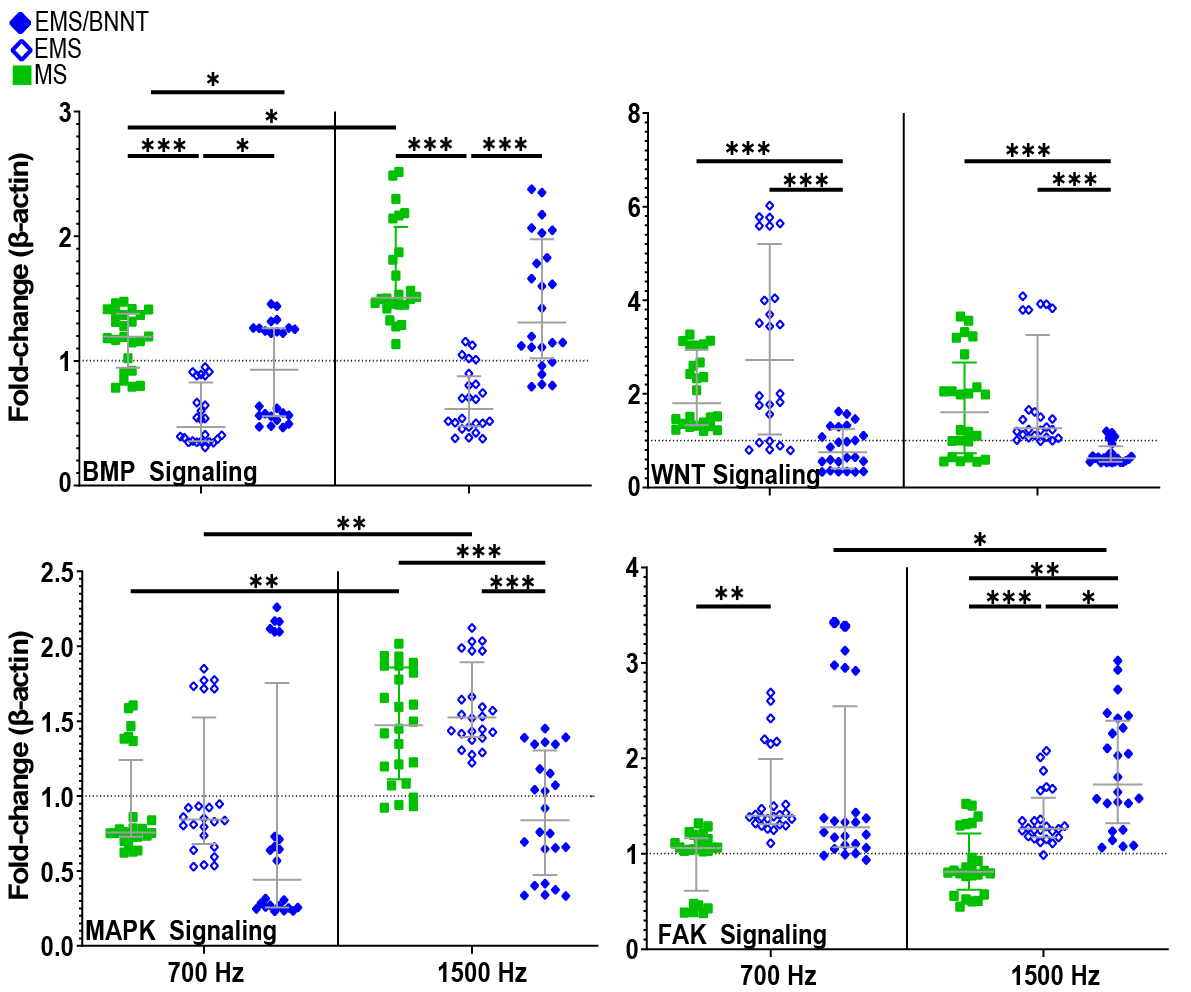


**Figure S11.** Comparison between 700 Hz and 1500 Hz Frequency Stimulation on hTDCS differentiation under MS, EMS or EMS/BNNT at day 5 . (***p<0.001, **p<0.01 ; *p<0.05 to respect to static control) Data is presented as Median ± IQR (N=4). (***p<0.001, **p<0.01 ; *p<0.05; to respect to static control).

**a**

**
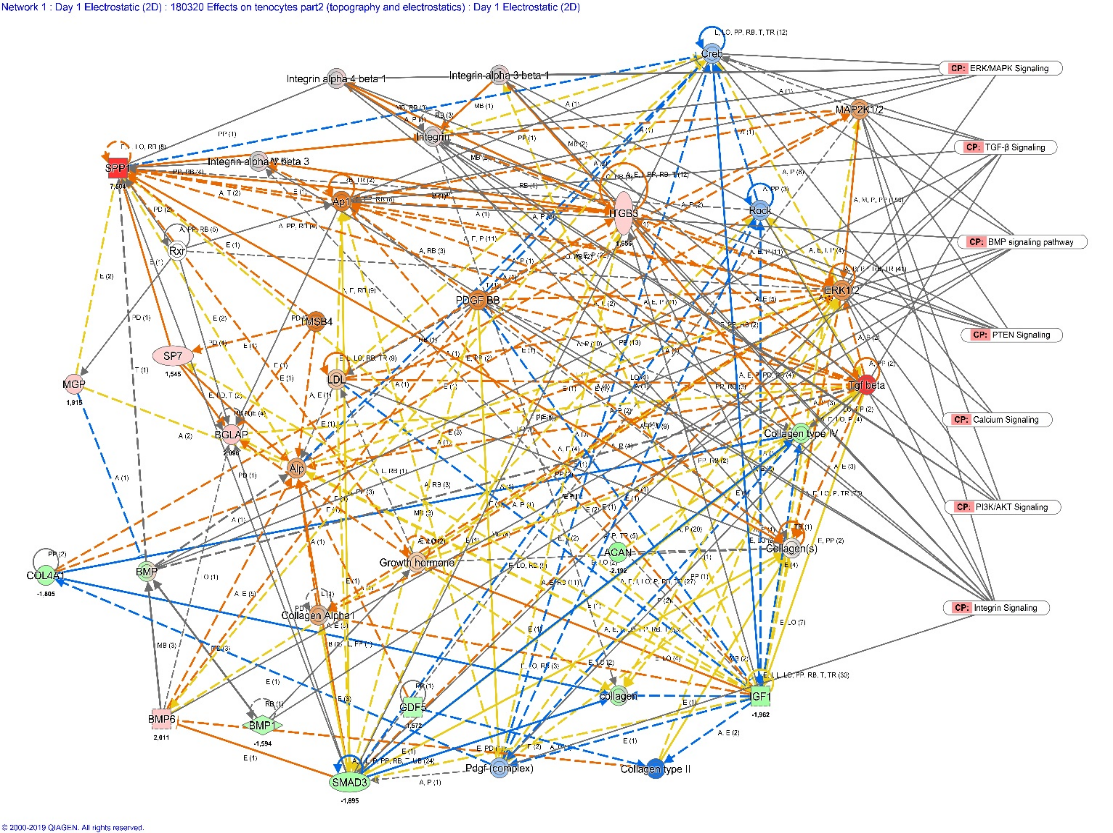
**

**b**


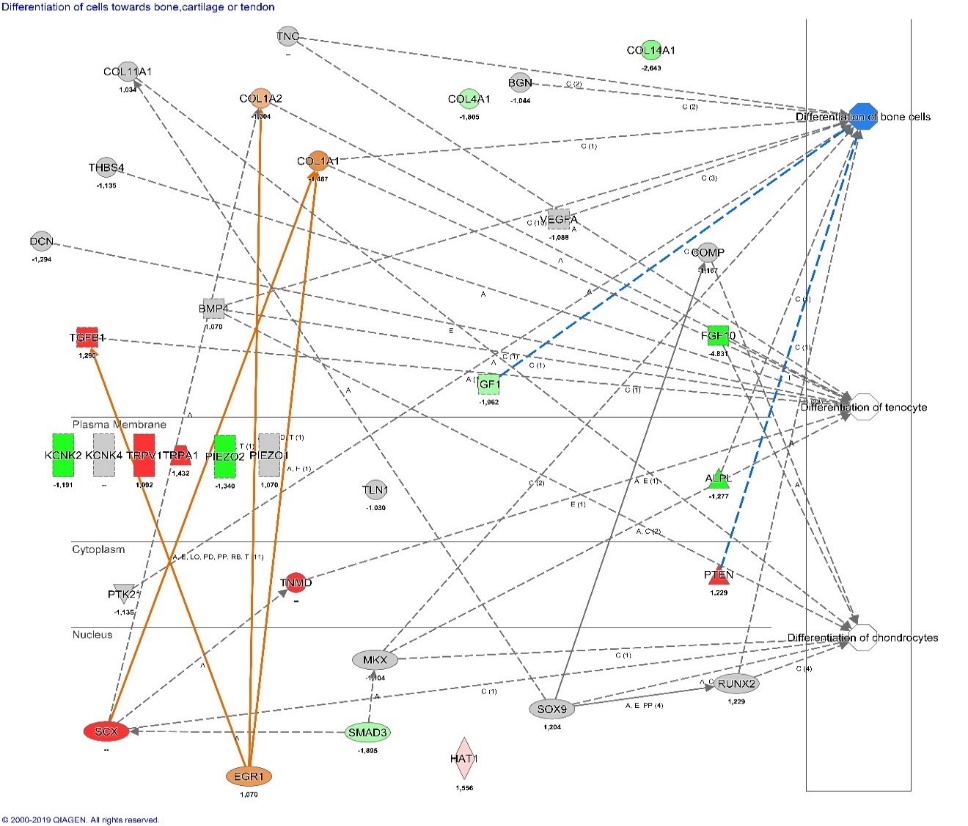


**Figure S12**. The network for tendon cells on piezoelectric surfaces demonstrates the mainly predicted biochemical upregulation of these pathways. An array (n=3) of different genes (96 genes) was used to evaluate the differences between cells cultured on piezoelectric or non-piezoelectric materials. The following figure shows the gene network generated in IPA using predictive biochemical interactions that link gene changes to signaling pathways, such as ERK and the canonical TGF-β/BMP signaling pathway. The analysis using IPA software showed that TGF-β (p-value=1.41x10-13) was an upstream regulator. The canonical pathways activated were related to TGF- β /BMP signaling and adhesion signaling pathways, including integrin, FAK, and paxillin.d) a) Signaling pathway associated with cellular differentiation processes towards bone, cartilage, or tendon formation under EMS.
